# Metabolic Profiling of Volatile Organic Compounds (VOCs) Emitted by the Pathogens *Francisella tularensis* and *Bacillus anthracis* in Liquid Culture

**DOI:** 10.1038/s41598-020-66136-0

**Published:** 2020-06-09

**Authors:** Kristen L. Reese, Amy Rasley, Julie R. Avila, A. Daniel Jones, Matthias Frank

**Affiliations:** 1 Biosciences and Biotechnology Division, Lawrence Livermore National Laboratory, 7000 East Avenue, Livermore, CA 94550 USA; 2 Department of Chemistry, Michigan State University, 578 S Shaw Lane, East Lansing, MI 48824 USA; 3 Department of Biochemistry and Molecular Biology, Michigan State University, 603 Wilson Rd, East Lansing, MI 48823 USA

**Keywords:** Metabolomics, Microbiology techniques, Biological techniques, Microbiology, Biomarkers

## Abstract

We conducted comprehensive (untargeted) metabolic profiling of volatile organic compounds (VOCs) emitted in culture by bacterial taxa *Francisella tularensis (F. tularensis)* subspecies *novicida* and *Bacillus anthracis (B. anthracis)* Sterne, surrogates for potential bacterial bioterrorism agents, as well as selective measurements of VOCs from their fully virulent counterparts, *F. tularensis* subspecies *tularensis* strain SCHU S4 and *B. anthracis* Ames. *F. tularensis* and *B. anthracis* were grown in liquid broth for time periods that covered logarithmic growth, stationary, and decline phases. VOCs emitted over the course of the growth phases were collected from the headspace above the cultures using solid phase microextraction (SPME) and were analyzed using gas chromatography-mass spectrometry (GC-MS). We developed criteria for distinguishing VOCs originating from bacteria versus background VOCs (originating from growth media only controls or sampling devices). Analyses of collected VOCs revealed methyl ketones, alcohols, esters, carboxylic acids, and nitrogen- and sulfur-containing compounds that were present in the bacterial cultures and absent (or present at only low abundance) in control samples indicating that these compounds originated from the bacteria. Distinct VOC profiles where observed for *F. tularensis* when compared with *B. anthracis* while the observed profiles of each of the two *F. tularensis* and *B. anthracis* strains exhibited some similarities. Furthermore, the relative abundance of VOCs was influenced by bacterial growth phase. These data illustrate the potential for VOC profiles to distinguish pathogens at the genus and species-level and to discriminate bacterial growth phases. The determination of VOC profiles lays the groundwork for non-invasive probes of bacterial metabolism and offers prospects for detection of microbe-specific VOC biomarkers from two potential biowarfare agents.

## Introduction

The study and detection of volatile organic compounds (VOCs) originating from or interacting with organisms ranging from bacteria to humans have numerous applications in biology, environmental sciences, medicine, food industry, and national security. VOCs consist of low molecular mass carbon-containing compounds that have low boiling points and measurable vapor pressures at Standard Temperature and Pressure (20 °C, 1 atm)^1^. The rapid progression of research within the last decade into the study of such VOCs, termed “volatilomics” or”volatolomics” and highlighted in recent reviews^2–4^, indicates this field can provide a new wealth of information complementary to the existing “-omics” fields, in particular, metabolomics and exposomics.

The measurement of VOCs and non-volatiles in exhaled breath is becoming an important rapid and non-invasive diagnostic tool to assess human physiology and health as well as a diagnostic tool for infections and systemic disease^2,5–7^. VOC markers in exhaled breath are also being explored to assess human chemical pharmacokinetics and environmental exposures to drugs, toxic materials, chemical or biological agents and other illicit materials. In this context, *in vitro* systems are often used to explore human exposome, microbiome and disease pathogenesis biomarkers^8,9^. One under-explored area of human volatile analysis is the analysis of exhaled breath to detect biomarkers indicative of an individual’s exposure to biosecurity-relevant bacterial pathogens. However, human breath VOC profiles may be specific to an individual, influenced by one’s unique personal microbiome, external exposures, and immunological responses. VOCs derived from metabolic processes specific to bacterial taxa are hypothesized to occur in the breath of infected individuals, requiring differentiation of the chemical signatures from background substances in exhaled breath, those formed by the action of biological agents, and those resulting from human-microbe interactions. This study has focused on identification of VOCs emitted from actively growing bacterial agents *in vitro* (independent of host) and represents an important, albeit only a first step towards potential VOC-based detection of an infection by select agents.

Bacteria emit VOCs as major metabolic products during their growth cycles, many of which have important functions as signaling molecules to either neighboring bacteria or higher organisms^10^. Bacterial VOC profiles comprise complex mixtures containing diverse structural and chemical complexity. Monitoring of volatile emissions of bacteria has been facilitated by use of SPME-GC-MS, and extensive literature on bacteria related to food safety and hospital-acquired infections demonstrates how volatile chemical signatures can differentiate bacteria from various genera, species, and subspecies. Prior work into sampling volatiles of active bacterial growth has largely focused upon pathogens related to clinical settings, with common examples originating from the genera *Pseudomonas*^11^*, Staphylococcus*^11^*, Klebsiella*^12^, and *Mycobacterium*^13^. For example, Rees, *et al*.^12^ reported aliphatic 2-ketones as the most abundant VOCs produced by *Klebsiella pneumoniae*, a common Gram-negative human pathogen, with less-abundant compound classes including esters, benzene derivatives, heterocycles, and nitrogen-containing compounds. Chen *et al*.^14^ focused on the time-dependent VOC emissions of several foodborne pathogens, identifying long chain methyl ketones (2-heptanone, 2-nonanone, 2-undecanone) and alcohols (1-octanol, 1-decanol, 1-dodecanol) as markers of three Gram-negative species (*E. coli, S. flexneri*, and *S. enteritidis*), while 3-hydroxy-2-butanone was identified as a marker of two Gram-positive bacteria (*S. aureus* and *L. monocytogenes*).

At present, there is less knowledge about volatiles released from bacterial threat agents, which would be useful for detecting the presence of and distinguishing such threat agents. Horsmon and Crouse^15^ used thermal desorption tubes coupled to gas chromatography-mass spectrometry (GC-MS) to describe VOC profiles emitted by cultures of *Yersinia pestis (Y. pestis)*, the causative agent of plague, and several strains from the genus *Bacillus*. They showed that VOC profiles and relative abundances of individual compounds distinguished bacterial genera as well as species within the same genus. However, their study did not provide a comprehensive analysis of detected VOCs and only qualitative differences determined by inspection of the profiles were used to distinguish species or genera. Lonsdale *et al*.^16^ used colorimetric sensor arrays (CSAs) to differentiate volatiles in the headspace of *Y. pestis* and *B. anthracis* cultures, and reported high specificity, accuracy, and sensitivity to very low bacterial concentrations. However, individual biomarkers leading to the colorimetric changes were not identified, and signal response could have been influenced by the growth media utilized.

Two bacteria of concern to biosecurity and subjects of the work presented here are the aerobic, facultative intracellular pathogen *Francisella tularensis (F. tularensis)* and the obligate, endospore-forming pathogen *Bacillus anthracis* (*B. anthracis*). Both are classified by the Center for Disease Control (CDC) as Tier 1 Select Agents on the CDC category A Bioterrorism Agents list^17^. *F. tularensis*, the causative agent of the disease tularemia, has been isolated from more than 200 separate organisms, and several subspecies are known human pathogens. The bacterium is highly infectious and easily aerosolized, requiring as few as ten bacteria to cause infections^18^. *B. anthracis*, the causative agent of the disease anthrax, forms resilient spores that survive chemical treatments, heat, lack of nutrients, and radiation, and has previously been developed into a bioweapon^19^. Detection of volatile biomarkers specific to the presence and growth of *F. tularensis* or *B. anthracis* through headspace sampling would be an important step towards developing a non-invasive metabolomics tool for rapid diagnosis of their presence in the lungs of subjects exposed to a biological attack.

Prior studies aimed at identification and/or differentiation of metabolites from *F. tularensis* or *B. anthracis* have largely focused on measuring profiles of pre-selected molecular targets in whole cell extracts. In particular, fatty acids have been profiled using GC-MS following esterification. The Voorhees group distinguished strains of *F. tularensis, B. anthracis, Brucella* spp. *abortus, melitensis*, and *neotomae*, and *Yersinia pestis* through analysis of fatty acid methyl ester (FAME) profiles using pyrolysis mass spectrometry in combination with an in-situ thermal transesterification^20,21^. Fatty acids of carbon chains ranging from 12:0 to 24:1 were identified, and principal components analysis (PCA) of the fatty acid profiles discriminated bacterial species. Li et al. distinguished *Francisella tularensis* subspecies *novicida*, *Escherichia coli*, and *Bacillus subtilis* by derivatizing fatty acids to form trimethylsilyl esters^22^. However, these studies required whole bacteria and sample preparation that was destructive to the bacteria, precluding analysis of metabolite changes over time in an unperturbed culture.

Our work presented here focused on the *in vitro*, non-invasive, untargeted profiling of VOCs from cultures of *F. tularensis* subspecies *novicida* (*Ft novicida*) and *B. anthracis* Sterne (*Ba* Sterne), both risk group 2 (RG2) surrogates for more virulent species, and from *F. tularensis* subspecies *tularensis* SCHU S4 (*Ft* SCHUS4) and *B. anthracis* Ames (*Ba* Ames), two fully virulent, risk group 3 (RG3) organisms. (For descriptions of risk group (RG) classifications of infectious microorganisms and recommended biosafety level (BSL) for their handling see the U.S. Department of Health and Human Services guide on Biosafety in Microbiological and Biomedical Laboratories^23^ or the World Health Organization Laboratory Biosafety Manual^24^). This work involved solid phase microextraction (SPME) sampling during multiple phases of pathogen growth of bacteria grown in biosafety level 2 (BSL-2) and biosafety level 3 (BSL-3) laboratories, respectively, and analysis by GC-MS. This *in vitro* determination of VOC profiles lays the groundwork for non-invasive investigation of bacterial metabolism of such organisms and represents the first steps towards potential VOC-based detection of an infection by such agents.

## Results

**Figure 1:**
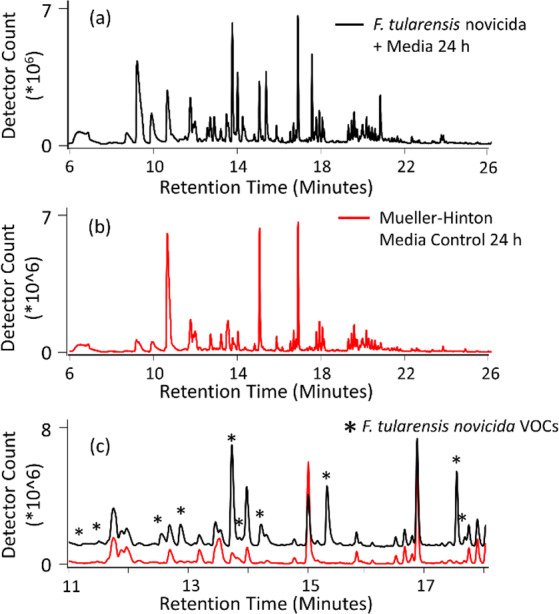
Examples of the chemical complexity exhibited by *F. tularensis novicida* cultures through comparison of the VOC total ion chromatograms at (**a**) 24 hours post inoculation, (**b**) corresponding Mueller-Hinton media control, and (**c**) overlay of both 1a and 1b over a smaller retention time range to emphasize chromatogram features, where stars indicate the bacteria-specific VOC emissions.

The complexity of chromatographic peaks detected through GC-MS analysis of each sample is illustrated by an observed profile of Ft *novicida* sampled 24 hours post-inoculation, representing the early stationary phase (Fig. 1a). However, many peaks originated from the Mueller-Hinton media and the SPME sampling device (Fig. 1b) and were considered background. Peaks representative of the bacterial signature were of lower relative abundance, highlighted on a smaller chromatographic scale in Fig. 1c. This complexity was similar for *Ft* SCHUS4 and both *B. anthracis* taxa (not pictured).

**Figure 2:**
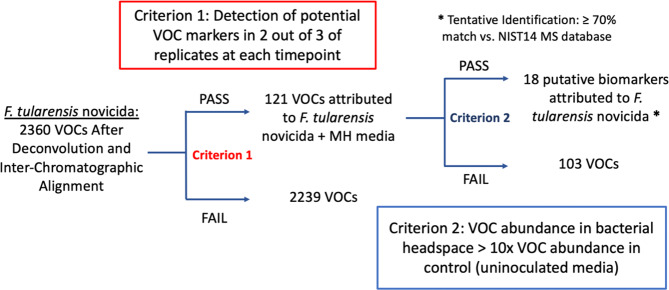
Example workflow of criteria utilized to filter list of detected VOCs to bacteria-specific biomarkers produced during growth, shown for *F. tularensis novicida*.

Detection of thousands of volatile compounds in the various cultures and timepoints for each of the taxa (Table 1) necessitated specified data-filtering criteria (see Methods) for quality control purposes. For example, more than 2000 VOCs were detected across all *Ft novicida* samples. Eliminating VOCs that did not appear in at least two of the triplicate measurements (Criterion 1) narrowed the dataset to 121 VOCs, a reduction of approximately 95% (Fig. 2). Further elimination of VOCs with relative abundances less than 10x the average relative abundance in the negative controls (Criterion 2) narrowed the dataset to 18 putative volatile biomarkers that were confidently attributed to *Ft novicida*. The same criteria were applied to the data from other bacterial species studied here, resulting in 38 putative VOC biomarkers for *Ft* SCHUS4, 30 biomarkers in *Ba* Sterne, and 56 biomarkers in *Ba* Ames (Table 1).

**Table 1:** Number of VOCs from *F. tularensis* and *B. anthracis* taxa detected by GC-MS and remaining after application of filtering criteria.

Species	Total VOCs Detected	VOCs Fail Criterion 1	VOCs Pass Criterion 1	VOCs Fail Criterion 2	VOCs Pass Criterion 2 (putative biomarkers)
*F. tularensis novicida*	2360	2239	121	103	18
*F. tularensis* SCHU S4	999	754	245	207	38
*B. anthracis* Sterne	1031	912	119	89	30
*B. anthracis* Ames	1022	745	277	221	56

### Results from RG2 species

Candidate bacterial VOC biomarkers from all timepoints were annotated through examination of both mass spectral library matching scores using the NIST14 database and experimental retention indices. Since all metabolite annotations in this report are based on comparisons to literature spectra and retention index values, they should be considered as satisfying confidence level 2 of the Metabolomics Standards Initiative recommendations for identification of compounds^25^. For *Ft novicida* (Table 2), 15 of the 18 biomarkers passed the set threshold of 70% match, while three were labeled as “unknowns” owing to poorer matches below that threshold. For *Ba* Sterne (Table 3), 18 of the 30 biomarkers passed the set threshold of 70% match, while the remainder were labeled “unknowns”. Inter-species diversity in emitted VOC biomarkers was observed. The *Ft novicida* profile contains odd-chain, aliphatic methyl ketones, alcohols, nitrogen-containing, and sulfur-containing volatiles. The *Ba* Sterne volatile profile is comprised of branched methyl ketones, followed by esters, carboxylic acids, alcohols, and sulfur-containing volatiles.

**Table 2:** Annotations of *F. tularensis novicida-*specific VOC markers through compound class, putative NIST ID, *m/z*, and retention index matching.

Class	Compound	MS Base Peak (*m/z*)	RI (Lit)	RI (Exp)	NIST 14 Match Factor
Alcohol	1-Butanol, 2-methyl-	70	739	719	74
Alcohol	2-Nonanol	45	1101	1113	80
Alcohol	Phenylethyl Alcohol	91	1116	1127	70
Alcohol	1-Nonanol	56	1173	1183	83
Alcohol	2-Undecanol	45	1308	1306	75
Methyl ketone	2-Heptanone	43	891	890	71
Methyl ketone	2-Nonanone	58	1092	1104	72
Methyl ketone	2-Undecanone	58	1294	1298	73
Methyl ketone	2-Tridecanone	58	1497	1492	93
Methyl ketone	2-Pentadecanone	58	1698	1690	87
Methyl ketone	58.0@26.267332	58	1902	1892	83
Nitrogen-containing	Pyrazine, 2,5-dimethyl-	108	917	911	80
Nitrogen-containing	2-Methyl-3-isopropylpyrazine	121	1056	1064	83
Sulfur containing	Dimethyl trisulfide	126	970	974	96
Sulfur containing	1-Propanol, 3-(methylthio)-	106	981	993	70
Unknown	*m/z* 121 _ RI 1002	121		1002	
Unknown	*m/z* 108 _ RI 1049	108		1049	
Unknown	*m/z* 133 _ RI 1110	133		1110

**Table 3:** Annotations of *B. anthracis* Sterne*-*specific VOC markers through compound class, putative NIST ID, *m/z*, and retention index matching.

Class	Compound	MS Base Peak (m/z)	RI (Lit)	RI (Exp)	NIST 14 Match Factor
Alcohols	4-Heptanol	55	872	893	86
Carboxylic Acid	Propanoic acid, 2-methyl-	43	772	744	70
Carboxylic Acid	Butanoic acid, 2-methyl-	74	861	865	90
Carboxylic Acid	Butanoic acid, 3-methyl-	60	863	853	86
Ester	Propanoic acid, 2-methyl-, butyl ester	89	898	961	97
Ester	Butanoic acid, butyl ester	71	995	1006	97
Ester	Butyl 2-methylbutanoate	103	1043	1053	89
Ester	Butanoic acid, 3-methyl-, butyl ester	85	1047	1058	83
Methyl Ketone	Methyl Isobutyl Ketone	43	735	719	73
Methyl Ketone	2-Hexanone, 5-methyl-	43	862	848	80
Methyl Ketone	2-Heptanone	43	891	889	94
Methyl Ketone	2-Heptanone, 6-methyl-	43	956	962	97
Methyl Ketone	2-Heptanone, 5-methyl-	43	971	973	73
Methyl Ketone	5-Hepten-2-one, 6-methyl-	108	986	977	70
Methyl Ketone	2-Heptanone, 4,6-dimethyl-	58	1045	1067	76
Sulfur containing compound	Butanethioic acid, S-methyl ester	43	874	834	78
Sulfur containing compound	Thiopivalic acid	85	959	945	71
Unknown	*m/z* 80 _ RI 715	80		715	
Unknown	*m/z* 57 _ RI 769	57		769	
Unknown	*m/z* 43 _ RI 791	43		791	
Unknown	*m/z* 43 _ RI 873	43		873	
Unknown	*m/z* 45 _ RI 901	45		901	
Unknown	*m/z* 57 _ RI 912	57		912	
Unknown	*m/z* 43 _ RI 956	43		956	
(methyl ketone)^a^	*m/z* 58 _ RI 962	58		962	
Unknown	*m/z* 43 _ RI 997	43		997	
Unknown	*m/z* 90 _ RI 1005	90		1005	
(methyl ketone)^a^	*m/z* 58 _ RI 1104	58		1104	
Unknown	*m/z* 83 _ RI 1145	83		1145	
(methyl ketone)^a^	*m/z* 58 _ RI 1554	58		1554	

^a^GC/MS fragmentation similar to observed methyl ketones

RI (Lit): Retention Index reported from NIST14

RI (Exp): Retention Index calculated from experiment.

**Figure 3:**
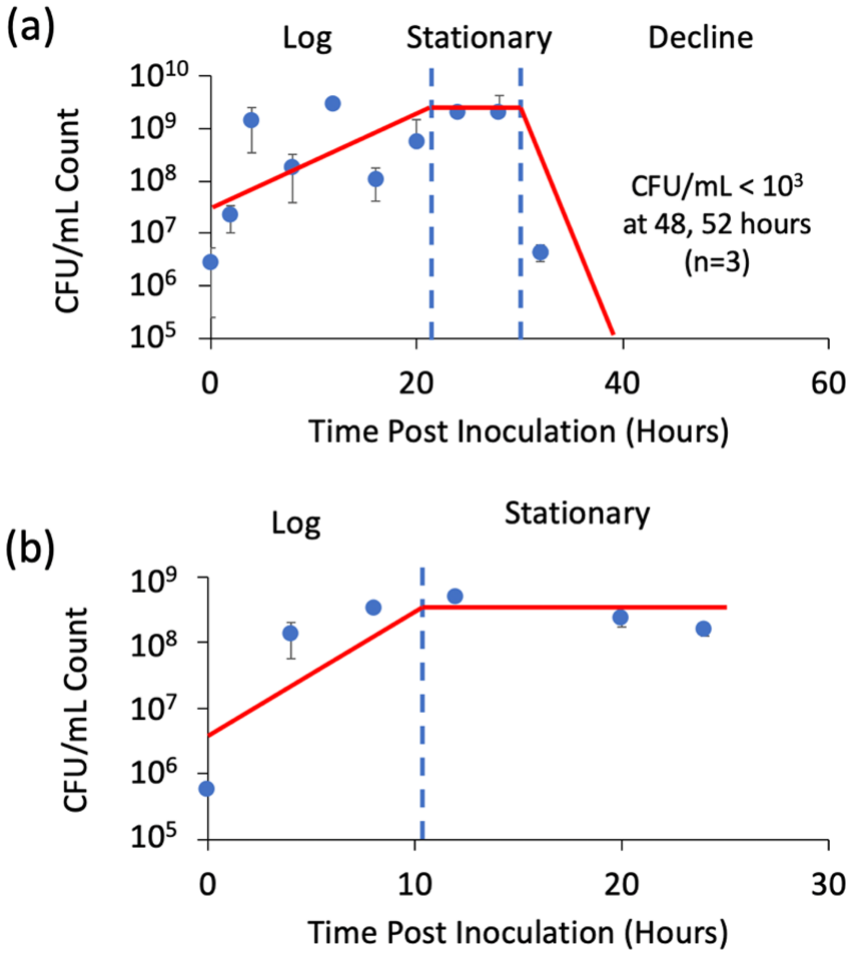
Growth curves of (**a**) *F. tularensis novicida* in modified Mueller-Hinton media over a 52-hour time period and (**b**) *B. anthracis* Sterne in Brain-Heart Infusion media over a 24-hour time period. Data points and error bars represent the means and standard deviations of CFU/mL determined from three culture replicates. Red curves represent trends in bacterial growth and indicator of growth phases.

Evaluation of potential markers requires assessment of the growth phase at each sampled timepoint post-inoculation of the culture flask. The logarithmic, stationary, and decline phases were identified based upon CFU measurements taken alongside SPME-VOC sampling. The data for both RG2 species, *Ft novicida* and *Ba* Sterne, are presented in Fig. 3. Logarithmic or “Log” phase, characterized by exponential bacterial growth, was observed to last for 20 hours and 8 hours, respectively. The bacterial counts rose approximately 3 orders of magnitude for both species, peaking at 1-2*10^9^ CFU/mL for *Ft novicida* and 5*10^8^ CFU/mL for *Ba* Sterne. For *Ft novicida* (Fig. 3a) the observed growth during that phase appeared rather variable. *Ft* cultures are known to be difficult to grow. Sampling more replicates may improve statistical confidence in future experiments. Stationary phase, occurring when the bacteria exhibit no additional growth due to a depleted nutrient source, was observed in both species. *Ba* Sterne measurements were completed at 24 hours post-inoculation while still in stationary phase. For *Ft novicida*, further growth phase changes were observed through a decline in viable bacterial growth to ~1*10^6^ CFU/mL at 32 h and no observable growth at the 48 h and 52 h post inoculation. The limit of detection for concentrations of viable bacteria was less than 1000 CFU/mL. Regardless, the CFU/mL counts were fairly consistent across triplicate measurements in both taxa, allowing assessments of growth phase.

**Figure 4:**
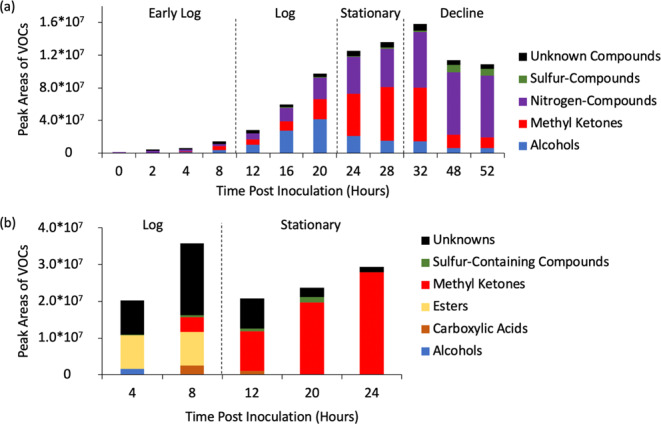
Mean combined peak areas (integrated detector counts) for marker compounds within individual classes at each timepoint post bacterial inoculation of cultures for (**a**) *F. tularensis novicida* and (**b**) *B. anthracis* Sterne.

The observed profiles of the VOC biomarkers varied with growth phase. The averaged relative abundances of biomarkers from *Ft novicida* and *Ba* Sterne are listed across all measured timepoints and grouped by compound class (Tables 4 and 5; Supplemental Tables 1 and 2). The mean combined chromatographic peak areas of each marker compound class measured at each time point for the two RG2 species are shown in Fig. 4. As can be seen in Fig. 4, the relative contributions for each of the marker compound classes to the total VOC biomarker signal evolve over time. Of the tentatively identified markers, chemical diversity was observed in the presence of ketones, aldehydes, alcohols, esters, carboxylic acids, nitrogen- or sulfur- containing markers, and alkanes. Although more biomarkers were detected for *Ba* Sterne (30) versus *Ft novicida* (18), the combined peak areas (total signal) of markers from *Ft novicida* at its peak growth (32 hours post-inoculation, stationary phase) were approximately 5x the total combined peak area of *Ba* Sterne VOCs at its peak growth (8 hours, logarithmic phase), attributed to the ~10x higher concentration of bacteria (compare Figs. 3 and 4). There was only a moderate correlation between combined marker peak areas and the bacteria concentration at any single timepoint. Peak areas and bacterial counts rose during the logarithmic phase for both species, but cumulative peak areas were stagnant or dropped during stationary phase despite bacterial concentration remaining steady.

**Table 4:** Average relative abundances (Log-10 Scale) of *F. tularensis novicida*-associated VOC markers at all measured timepoints, separated by growth phase.

Class	Time Post Inoculation (Hours)	0	2	4	8	12	16	20	24	28	32	48	52
	Growth Phase	Log Phase							Stationary Phase		Decline Phase		
	Compound	Abundance (Log 10 Values)											
Alcohols	1-Butanol, 2-methyl-	0.00	0.00	0.00	0.00	5.87^b^	5.84^b^	6.77	6.55^a^	6.77	6.76	5.70	5.44^b^
Alcohols	2-Nonanol	0.00	0.00	0.00	0.00	6.05	6.61	6.08	5.89	0.00	0.00	0.00	0.00
Alcohols	Phenylethyl Alcohol	0.00	0.00	5.66 a	6.07	6.29	6.36	6.54	6.75	6.74	6.81	6.67	6.67
Alcohols	1-Nonanol	0.00	0.00	5.80	6.35	6.70	7.26	7.46	6.98	6.43	6.24	5.90	5.85
Alcohols	2-Undecanol	0.00	0.00	0.00	0.00	6.14	6.35	6.27	6.13	5.96	0.00	0.00	0.00
Methyl Ketones	2-Heptanone	0.00	0.00	0.00	6.02	6.03 a	6.48	6.74	6.84	6.82	6.88	6.46	6.33
Methyl Ketones	2-Nonanone	0.00	0.00	0.00	6.44	6.60	6.84	7.11	7.31	7.41	7.48	6.96	6.85
Methyl Ketones	2-Undecanone	4.70 b,c	5.27 b,c	5.86	6.13	5.98	6.03	6.72	7.16	7.29	7.23	6.25	6.15
Methyl Ketones	2-Tridecanone	0.00	0.00	0.00	0.00	0.00	0.00	6.14	6.91	7.01	6.89	6.09	6.02
Methyl Ketones	2-Pentadecanone	0.00	0.00	0.00	0.00	0.00	0.00	4.96 b	6.23	6.50	6.51	6.03	6.04
Methyl Ketones	2-Heptadecanone	0.00	0.00	0.00	4.71 b	0.00	0.00	0.00	5.27	5.80	5.88	5.63	5.69
Nitrogen-Containing Compounds	Pyrazine, 2,5-dimethyl-	5.69 c	6.37 c	6.41 c	6.38 c	6.78 c	7.18 c	7.36	7.60	7.60	7.76	7.81	7.81
Nitrogen-Containing Compounds	2-Methyl-3-isopropylpyrazine	0.00	0.00	5.47 b	0.00	6.13	6.24	6.47	6.75	6.87	7.05	7.06	7.05
Sulfur-Containing Compounds	Dimethyl trisulfide	0.00	0.00	0.00	0.00	0.00	0.00	0.00	0.00	0.00	0.00	6.90	6.84
Sulfur-Containing Compounds	1-Propanol, 3-(methylthio)-	0.00	0.00	0.00	0.00	5.72 a	5.76 a	5.98	6.05	6.00	6.07	6.08	6.13
Unknown	*m/z* 121 _ RI 1002	0.00	6.14	5.71 b	6.10	5.90	5.79	5.86	6.15	6.18	6.33	6.16	6.09
Unknown	*m/z* 108 _ RI 1049	0.00	5.80	5.79	6.37	6.51 a	6.50 a	6.54 a	6.62	6.60	6.69	6.56	6.58
Unknown	*m/z* 133 _ RI 1110	0.00	0.00	0.00	0.00	0.00	0.00	5.07 b	6.07	6.01	6.08	5.94	5.87

Notes:

^a^VOC detected in 2/3 of triplicate measurements

^b^VOC detected in 1/3 of triplicate measurements

^c^VOC detected at levels less than 10x abundance in media blank.

**Table 5:** Average relative abundances (Log-10 Scale) of *B. anthracis* Sterne-associated VOC markers at all measured timepoints, separated by growth phase.

Class	Time Post Inoculation (Hours)	4	8	12	20	24
	Growth Phase	Log Phase		Stationary Phase		
	Compound	Abundance (Log 10 Values)				
Alcohols	4-Heptanol	6.15	0.00	0.00	0.00	0.00
Carboxylic Acid	Propanoic acid, 2-methyl-	0.00	5.38 a	5.53 a	0.00	0.00
Carboxylic Acid	Butanoic acid, 2-methyl-	0.00	5.93	5.31 a	0.00	0.00
Carboxylic Acid	Butanoic acid, 3-methyl-	0.00	6.16	5.68	0.00	0.00
Ester	Propanoic acid, 2-methyl-, butyl ester	6.50	6.65	0.00	0.00	0.00
Ester	Butanoic acid, butyl ester	6.70	6.22	0.00	0.00	0.00
Ester	Butyl 2-methylbutanoate	5.82	6.11	0.00	0.00	0.00
Ester	Butanoic acid, 3-methyl-, butyl ester	5.44	6.23	4.81 a	0.00	0.00
Methyl Ketone	Methyl Isobutyl Ketone	0.00	0.00	5.71	6.22 a	6.14
Methyl Ketone	2-Hexanone, 5-methyl-	0.00	5.43 a	6.05	6.52	6.76
Methyl Ketone	2-Heptanone	5.12 a	6.58	6.65	6.69	6.89
Methyl Ketone	2-Heptanone, 6-methyl-	0.00	0.00	6.62	6.92	7.02
Methyl Ketone	2-Heptanone, 5-methyl-	0.00	0.00	5.54 a	6.03	6.25
Methyl Ketone	5-Hepten-2-one, 6-methyl-	0.00	0.00	0.00	4.34	5.49
Methyl Ketone	2-Heptanone, 4,6-dimethyl-	0.00	0.00	4.80 b	5.26 a	5.64
Sulfur containing compound	Butanethioic acid, S-methyl ester	0.00	5.35	5.57	5.89	0.00
Sulfur containing compound	Thiopivalic acid	4.60 b	5.46	5.47	5.86	4.34 b
Unknown	*m/z* 80 _ RI 715	5.08 b,c	5.80	5.68 a	5.82	5.20 b
Unknown	*m/z* 57 _ RI 769	0.00	0.00	4.54 b	5.19	0.00
Unknown	*m/z* 43 _ RI 791	6.93	7.23	6.83	6.15	5.64 a
Unknown	*m/z* 43 _ RI 873	0.00	0.00	0.00	4.34 b	4.86
Unknown	*m/z* 45 _ RI 901	0.00	0.00	5.16 a	5.30	0.00
Unknown	*m/z* 57 _ RI 912	5.64	5.82	5.06 b	0.00	0.00
Unknown	*m/z* 43 _ RI 956	5.18	0.00	0.00	0.00	0.00
(methyl ketone)^a^	*m/z* 58 _ RI 962	0.00	5.82	0.00	0.00	0.00
Unknown	*m/z* 43 _ RI 997	0.00	4.88 b	0.00	5.03 a	5.18 a
Unknown	*m/z* 90 _ RI 1005	0.00	0.00	5.22	5.00 a	0.00
(methyl ketone)^a^	*m/z* 58 _ RI 1104	5.62	5.79	5.67	5.13 b	5.79
Unknown	*m/z* 83 _ RI 1145	0.00	5.11	4.57 a	0.00	0.00
(methyl ketone)^a^	*m/z* 58 _ RI 1554	0.00	4.54 b	5.00	0.00	0.00

Notes:

^a^VOC detected in 2/3 of triplicate measurements

^b^VOC detected in 1/3 of triplicate measurements

^c^VOC detected at levels less than 10x abundance in media blank

^d^GC/MS fragmentation similar to observed methyl ketones.

The biomarker peak areas for *Ft novicida* steadily increased throughout the logarithmic and stationary phases before decreasing during the decline phase. Alcohols steadily rose in relative abundance throughout the log phase and were dominant in the early log and log phases. While some alcohols persisted throughout the entire study, several were fully depleted at the longest timepoints measured (see 2-nonanol and 2-undecanol). Linear, odd-chain methyl ketones (or 2-ketones) were present throughout all growth phases, with ketones consisting of more than 13 carbons (longer than 2-tridecanone) being present only in the stationary phase and beyond. The contribution of methyl ketones peaked in stationary phase growth, and their decrease in the decline phase lowered total VOC relative abundances. Nitrogen-containing markers were present throughout the analysis of *F. tularensis* species due to the presence of 2,5-dimethylpyrazine, a marker that was a component of the growth media. However, the signal emitted from the bacterial cultures first exceeded 10x the signal in the media control at the 20-hour timepoint, prompting its inclusion as a potential *F. tularensis* marker. Combined with the signal from 2-methyl-3-isopropylpyrazine, nitrogen-containing markers comprised almost 70% of the chemical profile for the decline phase. Finally, *Ft novicida* noticeably displayed a large signal of dimethyltrisulfide as an abundant marker in the decline phase, comprising 7-8.5% of the total VOC signal.

Biomarker areas for *Ba* Sterne also changed dependent on growth phase, though fewer timepoints were measured compared to *Ft novicida*. Esters, carboxylic acids, and alcohols comprised a significant portion of the logarithmic phase VOC marker profiles. Esters were based on butanoic or propanoic acids, with methyl groups at the 2 or 3-carbon positions. Two carboxylic acids were also based on butanoic and propanoic acids, both methylated at the 2-carbon position. Alcohols were only present in the early log phase. The non-detection of these markers during the stationary phases (with the exception of 2-methyl-propanoic acid in early stationary phase) suggests use as precursors for further synthesis. Relative abundances of methyl ketones significantly increased during stationary phase. Moreover, while *Ft novicida* was dominated by straight-chain aliphatics, the methyl ketones in *Ba* Sterne contained methyl and aromatic substituents.

**Figure 5:**
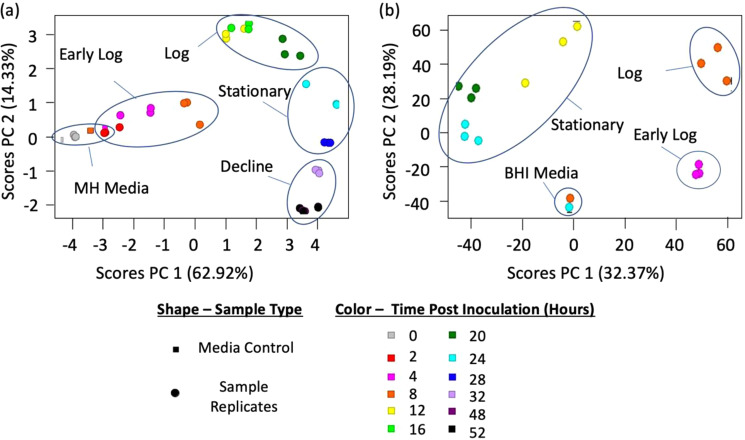
PCA scores plots for VOC marker profiles of (**a**) *F. tularensis novicida* and (**b**) *B. anthracis* Sterne generated using the peak areas of pathogen biomarkers across all timepoints. Each plot point represents one sample. Distinct chemical profiles were observed amongst labeled growth phases. Corresponding loadings plots to explain placement of samples are located in Supplemental Fig. 1.

Levels of VOC biomarkers for *Ft novicida* and *Ba* Sterne were subjected to principal component analysis (PCA) to visualize VOC profiles observed at different growth phases. The scores plots in Fig. 5a,b show clustering of all three culture replicates of the respective strains. The loadings plots, depicting the relative importance of individual markers towards sample positioning on PCs 1 and 2, are described in greater detail in Supplemental Fig. 1a,b. PCA groupings similarly exhibited distinct groupings of timepoints into clusters as determined in Fig. 4a,b for each species. The PCA scores plot provides additional verification of similarity of VOCs from culture replicates - profiles from each timepoint (same color) were positioned more closely to each other than to replicates of an adjacent timepoint, demonstrating fairly reproducible VOC marker profiles.

### Results from RG3 species

Determination of bacterial concentrations and VOC sampling of the RG3 pathogens grown in our BSL-3 laboratory were performed at select time points, as shown in Supplemental Table 3 for both *Ft* SCHUS4 and *Ba* Ames. *Ba* Ames exhibited growth throughout 24 hours, with bacterial concentrations rising to 2.5*10^7^ CFU/mL at the 24 h time point. Meanwhile *Ft* SCHUS4 concentration remained stagnant around 6.7*10^5^ CFU/mL throughout 24 hours of culture, hypothesized to remain in a lag phase after inoculation.

The decontamination protocols developed for our work in the BSL-3 laboratory on both *Ft* SCHUS4 and *Ba* Ames included wiping the SPME fiber exterior casing using bleach (see Supplemental Information). There was a potential for VOCs adsorbed on the internal fibers to be inadvertently oxidized. However, comparison of the VOC profiles from *B. anthracis* taxa obtained using the BSL-2 protocol without bleach wiping and the BSL-3 protocol that included the bleach wiping revealed a range of similar markers and/or compound classes, with no evidence of oxidized by-products for the profiles obtained using the BSL-3 protocol.

The VOC marker profile of *Ba* Ames displayed similarities to its RG2 counterpart *Ba* Sterne and is detailed in Table 7, where 18 of the 56 putative markers were identified. The VOC marker profile at the 6-hour timepoint of *Ba* Ames resembled the logarithmic VOC marker profile of *Ba* Sterne. Esters were the most abundant identified markers, consisting of propanoic and butanoic acid esters. Five esters were shared between both *B. anthracis* taxa. Subsequent compound classes included methyl ketones and carboxylic acids. Conversely, the VOC marker profile at the 24-hour timepoint of *Ba* Ames more closely resembled the stationary VOC marker profile of *Ba* Sterne. Methyl ketones were the dominant markers, while all esters have been depleted. Four methyl ketones were shared between *B. anthracis* taxa. Principal component analysis (PCA) of the level of VOC biomarkers was also applied to *Ba* Ames to visualize VOC profiles at different growth phases. Similar to *Ba* Sterne, it appears that different chemical profiles can be associated with different growth phases of *Ba* Ames (not shown here), however, more data points would be needed to draw stronger conclusions.

Conversely, the profile of VOCs from *Ft SCHUS4* (Table 6) had fewer similarities with *Ft novicida*. The majority of putative markers for *Ft SCHUS4* were classified as unknowns, with only 5 markers passing the conservative identification criteria. While none of the observed compounds passing the filtering criteria were shared between either species, the 6 and 24-hour timepoints both contained alcohols such as 4-methyl-3-heptanol and 1-dodecanol. Alcohols were also the dominant class of the logarithmic phase for *Ft novicida*. The lack of compound class similarities for determined markers could result from genetic differences between *Ft* SCHUS4 and *Ft novicida*. However, in agreement with the bacterial concentration data shown in Supplemental Table 3, the CFU counts suggests that *Ft SCHUS4* remained in a lag phase or a very early logarithmic phase throughout the first 24 h after inoculation. Additional measurements of the growth phases of *Ft* SCHUS4 over longer time periods are needed for a more comprehensive comparison of *Ft* SCHUS4 VOC markers against those of *Ft novicida*.

**Table 6:** Annotations of *F. tularensis* SCHU S4-specific VOC markers and average relative abundances (n = 3) at 6 and 24 hours post inoculation.

Class	Compound	MS Base Peak (*m/z*)	RI (Lit)	RI (Exp)	NIST14 Match Factor	Abundance (Log10 Values)	
						6 Hours	24 Hours
Alcohol	3-Heptanol, 4-methyl-	43	915	831	75	5.49 b	5.85
Alcohol	1-Dodecanol	55	1473	1469	82	0.00	5.77 a
Alcohol	Phenol, 2,5-bis(1,1-dimethylethyl)-	191	1514	1508	76	4.59 b	4.87 a
Aldehyde	Furfural	96	833	818	73	4.77 b	4.94 a
Nitrogen-containing compound	Pyrazine, methyl-	94	801	802	76	0.00	5.43 a
Unknown	*m/z* 41 _ RI 672	41		672		0.00	5.65 a
Unknown	*m/z* 133 _ RI 913	133		913		0.00	7.42
Unknown	*m/z* 57 _ RI 941	57		941		5.30	0.00
Unknown	*m/z* 43 _ RI 986	43		986		4.81 a	0.00
Unknown	*m/z* 105 _ RI 990	105		990		4.81 b	5.23
Unknown	*m/z* 207 _ RI 998	207		998		0.00	5.46 a
Unknown	*m/z* 121 _ RI 1010	121		1010		5.30	0.00
Unknown	*m/z* 69 _ RI 1034	69		1034		5.25 a	4.89 b
Unknown	*m/z* 43 _ RI 1063	43		1063		4.79 b	4.93 a
Unknown	*m/z* 71 _ RI 1185	71		1185		0.00	5.02 a
Unknown	*m/z* 57 _ RI 1199	57		1199		0.00	4.96 a
Unknown	*m/z* 57 _ RI 1303	57		1303		5.00 a	4.74 b
Unknown	*m/z* 119 _ RI 1365	119		1365		0.00	4.91 a
Unknown	*m/z* 57 _ RI 1370	57		1370		4.90	0.00
Unknown	*m/z* 57 _ RI 1398	57		1398		5.58 a	5.55 a
Unknown	*m/z* 69 _ RI 1527	69		1527		5.36	0.00
Unknown	*m/z* 40 _ RI 1536	40		1536		4.74 a	0.00
Unknown	*m/z* 163 _ RI 1667	163		1667		5.02 a	0.00
Unknown	*m/z* 71 _ RI 1810	71		1810		4.23 b	4.61 a
Unknown	*m/z* 40 _ RI 1972	40		1972		4.50 a	0.00
Unknown	*m/z* 73 _ RI 2363	73		2363		5.17 a	5.56
Unknown	*m/z* 73 _ RI 2520	73		2520		5.00 a	4.56 b
Unknown	*m/z* 73 _ RI 2521	73		2521		4.80 b	5.31 a
Unknown	*m/z* 73 _ RI 2679	73		2679		4.58 b	5.09 a
Unknown	*m/z* 96 _ RI 2777	96		2777		4.34 a	0.00
Unknown	*m/z* 208 _ RI 3117	208		3117		5.52 a	0.00
Unknown	*m/z* 207 _ RI 3154	207		3154		4.85 b	5.63 a
Unknown	*m/z* 97 _ RI 3306	97		3306		4.94 a	5.11
Unknown	*m/z* 207 _ RI 3356	207		3356		5.16 a	0.00
Unknown	*m/z* 97 _ RI 3393	97		3393		0.00	4.88 a
Unknown	*m/z* 97 _ RI 3425	97		3425		0.00	4.88 a
Unknown	*m/z* 97 _ RI 3548	97		3548		4.63 b	4.95 a
Unknown	*m/z* 208 _ RI 3572	208		3572		0.00	4.84 a

Notes:

^a^VOC detected in 2/3 of triplicate measurements

^b^VOC detected in 1/3 of triplicate measurements

RI (Lit): Retention Index reported from NIST14

RI (Exp): Retention Index calculated from experiment.

**Table 7:** Annotations of *B. anthracis* Ames-specific VOC markers and average relative abundances (n = 3) at 6 and 24 hours post inoculation.

Class	Compound	MS Base Peak (*m/z*)	RI (Lit)	RI (Exp)	NIST14 Match Factor	Abundance (Log10 Values)	
						6 Hours	24 Hours
Alcohol	3-Octanol, 3,6-dimethyl-	73	1043	1110	83	5.96 b	6.03 a
Carboxylic Acid	Butanoic acid, 2-methyl-	74	861	844	89	5.73	0.00
Ester	Acetic acid, butyl ester	43	812	794	96	7.29	0.00
Ester	Propanoic acid, 2-methyl-, butyl ester	89	898	964	97	7.03	0.00
Ester	Butanoic acid, butyl ester	71	995	1009	80	5.95	0.00
Ester	Butyl 2-methylbutanoate	103	1043	1055	97	6.48	0.00
Ester	Butanoic acid, 3-methyl-, butyl ester	85	1047	1060	95	6.40	0.00
Ester	2-Butenoic acid, 2-methyl-, 2-methylpropyl ester, (E)-	101	1112	1146	74	5.36	0.00
Ketone	3-Octanone	43	986	999	71	5.28	5.31
Methyl Ketone	Acetoin	45	713	691	85	6.86	0.00
Methyl Ketone	Methyl Isobutyl Ketone	43	735	712	89	0.00	6.39
Methyl Ketone	2-Pentanone, 3-methyl-	43	752	722	77	5.29 a	5.84
Methyl Ketone	2-Hexanone, 5-methyl-	43	862	851	94	0.00	6.64
Methyl Ketone	2-Heptanone	43	891	892	96	6.28	6.67
Methyl Ketone	2-Heptanone, 6-methyl-	43	956	965	93	0.00	7.16
Methyl Ketone	2-Nonanone	58	1092	1069	86	0.00	5.97
Methyl Ketone	Benzyl methyl ketone	91	1110	1141	85	0.00	5.67
Nitrogen-Containing Compound	Pyrazine, tetramethyl-	136	1089	1099	77	4.85 b	5.80
Unknown	*m/z* 42 _ RI 748	42		748		0.00	5.29
Unknown	*m/z* 40 _ RI 874	40		874		5.01 a	0.00
Unknown	*m/z* 42 _ RI 912	42		912		7.18 b	7.67
Unknown	*m/z* 57 _ RI 915	57		915		5.68 a	0.00
Unknown	*m/z* 43 _ RI 938	43		938		5.15 a	0.00
Unknown	*m/z* 93 _ RI 939	93		939		4.63 b	5.04 a
Unknown	*m/z* 43 _ RI 976	43		976		4.95 a	6.26
Unknown	*m/z* 57 _ RI 1012	57		1012		5.89 a	0.00
Unknown	*m/z* 69 _ RI 1034	69		1034		5.41 a	0.00
Unknown	*m/z* 43 _ RI 1054	43		1054		0.00	5.23
Unknown	*m/z* 55 _ RI 1057	55		1057		0.00	5.36
Unknown	*m/z* 43 _ RI 1063	43		1063		0.00	5.00 a
Unknown	*m/z* 57 _ RI 1071	57		1071		4.60 b	4.99 a
Unknown	*m/z* 130 _ RI 1126	130		1126		0.00	5.40 a
Unknown	*m/z* 149 _ RI 1169	149		1169		5.41 a	0.00
Unknown	*m/z* 57 _ RI 1180	57		1180		5.21	5.31
Unknown	*m/z* 91 _ RI 1189	91		1189		0.00	5.29
Unknown	*m/z* 55 _ RI 1201	55		1201		5.26	0.00
Unknown	*m/z* 108 _ RI 1205	108		1205		4.42 b	4.83 a
Unknown	*m/z* 339 _ RI 1218	339		1218		4.32 b	4.65 a
Unknown	*m/z* 71 _ RI 1285	71		1285		4.56 b	4.72 a
Unknown	*m/z* 119 _ RI 1365	119		1365		0.00	4.85 a
Unknown	*m/z* 401 _ RI 1564	401		1564		0.00	4.96 a
Unknown	*m/z* 405 _ RI 1685	405		1685		0.00	4.66 a
Unknown	*m/z* 40 _ RI 1747	40		1747		0.00	4.36 a
Unknown	*m/z* 40 _ RI 2271	40		2271		4.46 a	0.00
Unknown	*m/z* 73 _ RI 2521	73		2521		0.00	5.09 a
Unknown	*m/z* 281 _ RI 2674	281		2674		0.00	5.07 a
Unknown	*m/z* 281 _ RI 2983	281		2983		5.39 a	4.66 b
Unknown	*m/z* 281 _ RI 3008	281		3008		5.54 a	0.00
Unknown	*m/z* 208 _ RI 3069	208		3069		5.86 a	5.43 b
Unknown	*m/z* 209 _ RI 3135	209		3135		5.31 a	0.00
Unknown	*m/z* 207 _ RI 3154	207		3154		5.04 b	5.72
Unknown	*m/z* 281 _ RI 3173	281		3173		5.71 a	0.00
Unknown	*m/z* 281 _ RI 3190	281		3190		5.64 a	0.00
Unknown	*m/z* 208 _ RI 3439	208		3494		0.00	5.04 a
Unknown	*m/z* 97 _ RI 3521	97		3521		0.00	4.87 a
Unknown	*m/z* 97 _ RI 3533	97		3533		0.00	4.91 a

Notes:

^a^VOC detected in 2/3 of triplicate measurements

^b^VOC detected in 1/3 of triplicate measurements

RI (Lit): Retention Index reported from NIST14

RI (Exp): Retention Index calculated from experiment.

## Discussion

The methodology and results described here provide initial groundwork for detection and identification of volatile biomarkers from bacterial pathogens including fully virulent RG3 strains. The application of this non-invasive methodology for VOC profiling applied to actively growing *F. tularensis* and *B. anthracis* bacterial cultures revealed dynamic profiles, influenced by both the bacterial growth phase and bacterial concentration. At any given timepoint, isolation of the bacterial biomarkers was complicated by background volatiles, and data processing was applied uniformly across all sample types to identify bacterial biomarkers.

As we discuss the VOC profiles observed here, one should keep in mind that measured VOC profiles are influenced by the sampling and detection methods used. For example, the type of sorbent material used can introduce a sampling bias (sampling efficiency is dependent on partition behavior of each compound) and the sampling time and mass spectral analysis method influences the sensitivity with which compounds can be detected. Generally, detection limits for the basic type of SPME-GC-quadrupole MS used here for untargeted analysis (scan, not select ion mode) are on the order 1 ng of a compound injected into a column. It is conceivable that we have detected only the most prevalent VOCs and a larger number of relevant VOC biomarkers may be found if more efficient sampling techniques (e.g. thermal desorption tubes) and more sensitive mass spectrometry protocols, such as selected ion monitoring, are used. We explicitly note that absolute VOC quantification was not attempted here. For various practical reasons, absolute quantification of VOCs in entire cultures is challenging owing the desired use of stable isotope-labeled internal standards that are susceptible to metabolic degradation. Also, our cultures were not fully enclosed (we used flasks with vented caps) because they required gas exchange (oxygen) to sustain growth and needed to be vented to avoid buildup of pressure. However, relative abundances of compounds were compared among cultures by integrating chromatographic peaks across species and timepoints.

The cumulative VOC profiles of *Ft novicida*, *Ft* SCHUS4, *Ba* Sterne, and *Ba* Ames determined here included representatives of different compound classes such as methyl ketones, alcohols, nitrogen-containing compounds, sulfur-containing compounds, carboxylic acids, esters, and various unidentified biomarkers. Exhaustive identification of every pathway that produces these volatiles is beyond the scope of this discussion, but several likely routes of biosynthesis are enumerated below.

### Ketones

Ketones were abundant markers, present in all pathogens except *Ft* SCHUS4, and largely as methyl ketones. The methyl ketones are likely formed by modifying products of the fatty acid biosynthesis pathway, specifically the β-oxidation of fatty acids^26^. Odd-chain methyl ketones can be formed through the decarboxylation of even-carbon β-keto acids. Conversely, even-carbon methyl ketones arise from odd-carbon fatty acids and occur with lower frequency^27^.

Interestingly, methyl ketones with straight-chain alkane branches were abundant in *Ft novicida*, while primarily branched and aromatic methyl ketones were prevalent in *Ba* Sterne and Ames. This difference may stem from *B. anthracis* being Gram-positive, whereas *F. tularensis* is Gram-negative. Synthesis of fatty acids in Gram-positive and Gram-negative bacteria is controlled by enzymes with different preferred substrates. For example, comparison of the enzyme β-ketoacyl-acyl carrier protein synthase III from Gram-negative *E. coli* and Gram-positive bacteria *S. aureus* demonstrated a larger binding pocket in the Gram-positive bacteria, thus having a higher likelihood for synthesis of branched-chain alkyl substrates^28^. A smaller binding pocket for the Gram-positive bacteria would limit the use of branched-chain alkyl substrates.

### Alcohols

While alcohols were present for both *F. tularensis* and *B. anthracis*, their number and relative abundances were greater in *F. tularensis*. Alcohols may be synthesized from the breakdown products of β-oxidation of fatty acids, for example after enzymatic reduction of carboxylic acids^27,29^. The fatty acid chains observed for the alcohols class exhibited diversity, including straight-chain, branched chain, and aromatic substituents. 1-nonanol was likely formed by reduction of the fatty acid. The 2-alkanols (2-nonanol and 2-undecanol) are postulated to be derived from corresponding methyl ketones as reduced intermediates, since the corresponding methyl ketones were also detected at all timepoints where the 2-alkanols were detected, usually at a higher relative abundance. The aromatic alcohol phenylethyl alcohol is a widely occurring VOC produced by several bacterial species. Volatile alcohols have been shown to play a role in growth inhibition of several bacteria and fungi^30,31^.

### Sulfur-containing compounds

Dimethyltrisulfide was an abundant VOC uniquely present in *Ft novicida* during the decline phase, when no viable bacteria were detected. Sulfur-containing VOCs are attributed to breakdown of the amino acids cysteine and methionine^27^. Dimethyltrisulfide has previously been observed as product of human decomposition caused by bacteria.

### Nitrogen-containing compounds

The detection of nitrogen-containing pyrazine markers produced by bacteria is complicated by endogenous pyrazine VOCs present in the growth media^27^. The sterilization of growth media through autoclaving heats amino acids and reducing sugars, producing pyrazines via the Maillard Reaction^32^. The Mueller-Hinton growth media controls consistently produced 2,5-dimethylpyrazine, and a similar relative abundance was observed in the *Ft novicida* cultures through the first 16 h of growth. At 20 h of growth and beyond, the relative abundance of 2,5-dimethylpyrazine rose more than 10x the relative abundance of the controls, suggesting the growing bacteria have active involvement in biosynthesis of pyrazines. An additional pyrazine, 2-methyl-3-isopropylpyrazine, was also observed. Isopropyl substituents to pyrazine compounds are not common constituents in bacterial volatiles^27^. Therefore, we hypothesize both pyrazines originate from *Ft novicida* under the chosen growth conditions. Pyrazines have also been observed as volatile byproducts of bacterial metabolism, for example, in the genera *Streptomyces*^33^ and *Bacillus*^34^. Only one nitrogen-containing compound, tetramethylpyrazine, was observed in *Ba* Ames during the last observed timepoint, estimated to be in the logarithmic phase, but was not as abundant in as in *Ft novicida*. This is compounded by the different growth media utilized, which emphasizes the need for careful evaluation when comparing biomarkers across different growth conditions and species.

### Esters and carboxylic acid compounds

Esters and carboxylic acids were detected exclusively in both *Ba* Sterne and *Ba* Ames, but not in the *F. tularensis* strains. The identified *B. anthracis* markers contained either propanoic or butanoic acid as the backbone for methylated esters or the side-chain for carboxylic acids. The formation of esters and carboxylic acids can be derived from shared metabolic pathways occurring during normal bacterial growth, such as oxidation of fatty acids or amino acid metabolism. As the ester and carboxylic acid compounds were only observed during the logarithmic growth stage, this demonstrates a shift in *B. anthracis* metabolism once bacteria reach the stationary phase.

### Evidence of dynamic metabolic processes

For all four bacterial taxa studied here, their VOC marker profiles varied as function of time after inoculation/culture start and, as observed for *Ft novicida*, *Ba* Sterne and *Ba* Ames, varied distinctly across their growth phases. For *Ft novicida* and *Ba* Sterne, this was also shown through application of PCA, which produced distinct groupings for the VOC markers of different growth phases. For example, in the *Ft novicida* cultures, the methyl ketones, once produced, were present throughout the remainder of the experiment. However, select alcohols (2-nonanol and 2-undecanol) were not detected after a mid-stationary (28-hour) timepoint. This suggests alcohols were depleted in the liquid culture, potentially as precursors in ongoing bacterial metabolism. Once the basic metabolism of isolated pathogens is determined, changes in the marker profiles when additional variables are added (e.g. different substrates) can help drive inferences on metabolic activity of complex systems.

### Comparison of VOC markers for RG3 vs. RG2 strains

In comparing the putative VOC biomarkers identified for *Ba* Ames (RG3) to those for *Ba* Sterne (RG2), we found some similarities, but also distinct differences. In contrast, the profile of VOCs from *Ft* SCHU S4 (RG3) had fewer similarities with *Ft novicida* (RG2). The relative similarities between *Ba* Ames and *Ba* Sterne may stem from the close genetic relationship of these two strains (*Ba* Sterne is missing one of the two plasmids that *Ba* Ames has but is otherwise genetically very similar to *Ba* Ames^35^). In contrast, *Ft* SCHU S4 and *Ft novicida* are genetically more distinct^36^. If further confirmed in future studies, this may have implications for the use of RG2 “simulants” to develop sensors and algorithms for detecting exposures to the related RG3 pathogens. In the biodefense community, *Ba* Sterne is generally considered a good simulant for *Ba* Ames. For *Ft,* RG2 simulants other than *Ft novicida* may be considered.

Future VOC sampling should be performed on additional subspecies of *F. tularensis* (e.g., spp. holarctica) and *B. anthracis* (e.g., spp. Vollum or H9401) to investigate whether these profiles are unique to a subspecies, species, or bacterial pathogens in general. Several markers identified in this study have been previously reported as emissions of other bacterial types. For example, Chen *et al*.^14^ reported 2-heptanone, 2-nonanone, and 2-undecanone in *E. coli* but did not detect higher carbon methyl ketones. Rees *et al*.^12,13^ reported both even and odd-chain methyl ketones, including 2-hexanone, 2-heptanone, 2-nonanone, and 2-decanone products from *Klebsiella pneumoniae*, where the presence of even-chain methyl ketones suggests a different or complementary synthesis pathway for volatile production.

One of the long-term goals of our project seeks to use VOCs as breath-based diagnostic markers towards the detection of biowarfare agents in patients after a suspected biological attack. During a hypothetical pathogen infection in humans, the VOCs in breath may be derived from 1) the invading pathogen, 2) the human breath volatilome, or 3) interactions between the human host and pathogen. This study represents our first step in non-invasive methodology and data analysis optimization, extensively profiling two attenuated pathogen or RG2 species and screening their RG3 virulent counterparts in optimized growth media. The number of compounds identified in the human breath volatilome continues to grow through targeted and untargeted studies. A searchable database of breath-specific compounds in the “human volatilome” has been curated by the U.S. Environmental Protection Agency (EPA) and is continuously updated^37,38^. A survey of this list against the *F. tularensis* RG2 and RG3 profiles found here revealed 2-heptanone and 2-nonanone have been detected in human breath, while a comparison against the *B. anthracis* RG2 and RG3 profiles revealed 2-methylpropanoic acid, 2-heptanone, 6-methyl-2-heptanone, and 5-methyl-5-hepten-2-one that have been reported in human breath. Also, it is important to note that the volatiles in this database may not be commonly shared among all people, as human breath has been shown to be influenced by one’s unique personal microbiome, external exposures, and immunological responses. The effects of shared volatiles between pathogens and a human host must be evaluated in further studies that better simulate an *in vivo* infection, as well as identifying markers unique to that interaction.

Future work into “baseline” human breath signatures, a pathogen-specific volatilome, and host-microbe interactions are required for evaluation of VOCs as diagnostic tools for human health. Towards a pathogen-specific volatilome, further efforts include expanding both the number of bacterial species and evaluating the effects of chosen growth media on VOCs produced. Additionally, as animal model studies have established a low bacterial count can establish infections (e.g. 10 bacterial counts for *F. tularensis* in primate models), optimization of signal detection will also be investigated, as the conditions employed here used relatively high bacterial counts. Finally, future work should make the transition from *in vitro* studies into experiments more closely aligned with *in vivo* studies, such as initiating bacterial infection of human lung cell cultures and analyzing the resultant profiles for discovery of overlapping volatile compounds that may serve as diagnostic markers of human exposures to biosecurity-relevant pathogens.

## Conclusions

This study adapted a SPME-GC-MS methodology for noninvasive profiling of VOCs emitted from actively growing pathogens, specifically potential biowarfare bacterial agents and their surrogates, in both BSL-2 and BSL-3 settings. The devised methodology detected volatile biomarkers that were reflective of both the presence and physiological growth phase of pathogens. The data processing employed distinguished signals from the pathogens against a complex chemical background, in this case aided by the use of powerful software (MassHunter, MPP) for compound annotation and visualization of GC-MS data. Although the devised methodology based on SPME-GC-quadrupole MS does not represent the pinnacle of sensitivity, a number of relatively robust and reproducible putative volatile biomarkers could be detected. Further confirmation of these markers should be pursued in more repeat experiments across a wider range of growth conditions. More efficient VOC collection methods and more sensitive mass spectral analysis techniques may also uncover additional markers in the future.

Detection and identification of metabolites specific to taxa or species provides the first steps to understanding their formation via various metabolic pathways and the genetic basis for these pathways. We acknowledge that the work presented here constitutes only initial scoping experiments. While this work demonstrates the applicability of this method and found a number of interesting volatile biomarkers, this work needs to be expanded to determine the influence of various experimental factors on markers. We recommend that future research include determining the dependence of pathogen-produced volatiles on environmental conditions (e.g. chosen growth media) and use of different VOC collection methods (e.g. thermal desorption tubes) to achieve lower detection limits. Elucidation of comprehensive bacterial profiles is expected to provide clues about bacterial metabolism in controlled environments, which can further inform research into metabolic processes when pathogens are in other settings (i.e. a host). Ultimately, such biomarkers may yield useful information about metabolism in bacterial taxa and may facilitate new applications in biodetection. Distinct volatile profiles have potential to be used for the detection of pathogens in the context of biosecurity-relevant exposures of humans during a biological attack.

Results from this work have implications in the larger volatilomics community, both within the field of pathogens study and beyond. While volatile compounds from *B. anthracis* have been previously studied, this is the first study, to our knowledge, to profile volatile emissions of *F. tularensis*. Future databases can incorporate biomarker signatures from various pathogen species for means of relevant comparisons.

## Methods

### Strains and growth media

*F. tularensis* subspecies *novicida* (strain U112; RG2) and subspecies *tularensis* (strain SCHU S4; RG3) were obtained from the CDC and Brigham Young University, respectively. *B. anthracis* (strain Sterne; RG2) and Ames (strain Ames; RG3) were obtained from a collaborator at Dugway Proving Grounds. The agar plates and liquid growth media for bacterial growth were prepared separately for each species. Different media were chosen to achieve optimal bacterial growth. *F. tularensis* was grown using a modified Mueller-Hinton (MH) growth media^39^ (Becton Dickinson (BD) Difco, Franklin Lakes, NJ) supplemented with 0.1% glucose, 0.025% ferric pyrophosphate (Sigma-Aldrich, St. Louis, MO), and 0.02% IsoVitaleX (BD Difco); *B. anthracis* was grown with Brain-Heart Infusion (BHI) growth media (Becton Dickinson (BD) Difco, Franklin Lakes, NJ). RG2 strains and RG3 strains were grown, prepared, and sampled in a biosafety level 2 (BSL-2) laboratory and biosafety level 3 (BSL-3) laboratory, respectively.

### Preparation of bacterial headspace

Bacterial colonies were selected after overnight incubation on agar plates and transferred to 10 mL of liquid modified MH media or BHI media, respectively. Bacteria were cultured in media under aerobic conditions with overnight incubation at 37 °C and 170 rpm shaking. For each species and experiment, three 100-µL aliquots were inoculated into three separate 20 mL portions of fresh liquid media (1:200 dilutions) and incubated in three 250-mL disposable polycarbonate Erlenmeyer flasks with vented caps at 37 °C and 170 rpm shaking. The VOC profiles from the headspaces of each of the triplicate bacterial cultures (replicates) and the number of viable bacteria were sampled and assessed at multiple timepoints. In addition to the three replicates of each pathogen species, an uninoculated liquid media flask was simultaneously prepared and VOCs sampled from it as a negative (media-only) control.

### Sampling VOCs from bacterial headspace (RG2 strains)

The VOC profiles of bacterial headspaces and media controls were sampled at different time intervals depending on bacterial growth rates and experimental setups using a protocol developed here that some of the authors also applied for headspace analysis of algal cultures in other work^40^. *Ft novicida* cultures were sampled at the following timepoints: 0, 2, 4, 8, 12, 16, 20, 24, 28, 32, 48, and 52 hours post-inoculation. *Ba* Sterne cultures were sampled at the following timepoints: 0, 4, 8, 12, 20, and 24 hours post-inoculation. At the time of sample collection, Erlenmeyer flasks were removed from the incubator-shaker and transferred to a biosafety cabinet. Headspace VOCs were immediately collected for 30 minutes on a field-portable 2 cm solid-phase microextraction (SPME) fiber with a 65 µm polydimethylsiloxane/divinylbenzene (PDMS/DVB) coating (Supelco, Bellefonte, PA) with no agitation of the flask. At each timepoint, one unexposed SPME fiber (fiber remaining retracted behind the septum in the SPME housing) was placed within the biosafety cabinet where the SPME sampling of cultures was taking place. These fibers served as “travel blanks” to account for potential background volatiles leaking onto retracted fibers over time during storage or transportation to the GC-MS analysis laboratory. These “travel blanks” were analyzed concurrently with fibers exposed to cultures. After collection, all SPME fibers were stored in refrigerators at 2–4 °C until analysis. Data acquisition on the GC-MS occurred within 3 weeks of collection.

### Sampling VOCs from Bacterial Headspace (RG3 strains) and Transfer of SPME Samples to BSL-2 Facility

The VOC profiles of RG3 *Ft* SCHUS4 and *Ba* Ames as well as corresponding media controls were sampled at the following timepoints: 0, 6, and 24 hours post-inoculation. Timepoints were chosen to capture the exponential and stationary growth phase in each species. At the time of sample collection, Erlenmeyer flasks were removed from the incubator-shaker and transferred to a biosafety cabinet within the BSL-3 facility. Flasks were allowed to sit in the BSC for 30 minutes prior to sampling in order to allow any aerosols to settle. Headspace VOCs were collected for 30 minutes on SPME fibers with no agitation of the flask. After collection, SPME fiber devices were decontaminated by bleach wiping the entirety of their external housing for 1 min apiece, and residual bleach was removed via wiping. The process of bleach wiping to prevent accidental transfer of pathogens out of the BSL-3 facilities was tested and validated. The overall protocol was approved by the Institutional Biosafety Committee (IBC) at LLNL (see Supplemental Protocol in Supplemental Information). SPME fibers were transferred from the BSL-3 to BSL-2 facilities and stored in refrigerators at 2–4 °C until analysis, as previously described. In analyzing the samples collected in the BSL-3, we did not find any indication that bleach wiping may have altered the compounds detected e.g. by introducing chlorinated compounds.

### Determination of bacterial concentrations

The growth phase (logarithmic, stationary, decline) of each organism was estimated by monitoring the concentration of viable bacteria over the course of the experiment for all biological replicates. Aliquots (1 mL) of all bacterial cultures were collected immediately following VOC sampling at each of the timepoints, and the Erlenmeyer flasks were subsequently placed back into the incubator-shaker. The aliquots were serially diluted between 10^−2^ to 10^−7^ depending on expected growth phase. A preliminary experiment was performed by plating in duplicate 10-fold dilutions to determine the appropriate serial dilution for each growth phase. The dilution factor was selected to achieve a target concentration of 30-300 cells per plate for counting. Dilutions were plated in duplicate (100-μL aliquots) on agar plates to determine the number of colony-forming units (CFU). Bacterial concentrations are reported as CFU counts per mL of liquid culture.

### Data acquisition parameters

The data acquisition followed a procedure similar to the one previously described for algal VOCs and is briefly summarized here^40^. VOC analyses were performed on an Agilent 5975 T GC-MSD (Agilent Technologies, Santa Clara, CA) using an Agilent HP-5ms column (30 m x 250 µm x 0.25 µm) coupled to a single quadrupole mass analyzer with helium carrier gas at a constant flow rate of 1.2 mL/min. Volatiles absorbed by the SPME fiber were desorbed in the heated (250 °C) GC inlet for 60-seconds using splitless injection. The column temperature was programmed to start at 40 °C for 6 min, then heated at 8 °C/min from 40 to 280 °C and held for 4 min (total run time = 40 min). Ions were generated using electron ionization (EI) (70 eV) and acquired at 4 scans/s over *m/z* 35-450. Data acquisition was performed using ChemStation (version E.02.02). A commercial GC-MS reference standard (S-22329; AccuStandard, New Haven, CT) was used to evaluate day-to-day performance of the GC-MS system and to calculate retention indices.

### Data processing

After data acquisition, data processing procedures and criteria were applied to detect and identify taxa-specific biomarkers similar to the work previously described for algal VOCs^40^. All ChemStation data files (consisting of data from biological replicates, media controls, and travel fibers) were translated using MassHunter GC/MS Translator B.07.05 for compatibility with Agilent’s Mass Hunter Qualitative software (version B.07.00 SP2) and Mass Profiler Professional (MPP) 12.6.1 software. These programs enabled sophisticated organization of individual MS files into complex datasets for chemometric analyses.

Chromatographic deconvolution and visualization were performed using MassHunter Qualitative using a Retention Time window size factor of 90.0, signal-to-noise ratio threshold of 2.00, and absolute ion height filter of 1000 counts^40^. An arbitrary small value of 1 was assigned across all samples to the signal value for compounds that were not detected. Detected peaks were transferred into MPP and inter-aligned using a retention time tolerance of 0.15 minutes, mass spectral match factor of 0.6 (of maximum 1.0), and a delta *m/z* tolerance of 0.2 Da. Annotation of the aligned compounds was performed by searching spectra against the NIST14 mass spectral database. Compounds with mass spectral matches ≥70% were subsequently identified by the name of the match with the highest score. Identifications with literature retention indices deviating more than 5% from the experimental retention indices were rejected. Compounds that did not exceed the mass spectral match or retention index threshold were annotated using the base peak *m/z* and retention index (e.g. “Unknown *m/z* 121_RI 1002”).

The reported abundance values in this work are relative abundances of compounds, obtained by integrating the signal in their chromatographic peaks. Relative abundances are compared between different measurements (timepoints, species). Absolute quantification of VOCs in the headspace above bacterial cultures is challenging with our method. For example, our culture vessels were not fully enclosed due to the use of vented caps designed to facilitate gas exchange and avoid pressure buildups, and some loss of VOCs may have occurred. The retention of analytes is also affected by sorbent material, sampling time, and potential saturation, whereas the desorption is affected by extraction time and temperature. Some relative quantitation could be achieved using internal standards, whether pre-loaded or spiked into cultures, but also has a number of practical issues. Therefore, for the purposes of our work, absolute quantification was not attempted.

Two filtering criteria were used to identify relatively robust and reproducible VOCs as the most likely candidate compounds for potential taxa-specific biomarkers. The first criterion required detection of a potential biomarker in at least two of three culture replicates at a given sampling timepoint. This “2 out of 3 replicates” filter criterion was chosen as a compromise to require some level of reproducibility while also allowing for some biological variability that is often encountered in experiments involving live biological systems. Some of the detected compounds were present at fairly low concentrations and some biological variability could have easily pushed a compound below the detection threshold in one of the replicates. We chose this “2 out of 3” criterion in order to avoid missing some potentially interesting markers by applying too stringent a criterion. The second criterion concerned the presence or absence of a marker in a biological culture relative to the media and travel blank controls appropriate for each organism. A compound was removed from consideration as a potential candidate if its relative abundance in the biological replicates was less than ten times the relative abundance in the control.

The VOCs identified as putative taxa-specific biomarkers were compared with regard to both individual markers and groups of markers encompassing a compound class. First, the presence or absence of these markers in each growth phase (logarithmic, stationary, and decline) was determined. Second, the calculated peak areas of markers, also referred to as relative abundances here, were compared amongst biological replicates to assess consistency of detection. Finally, principal component analysis (PCA) was used as a dimension-reduction strategy to visualize covariance in the dataset. Only markers remaining after the filtering criteria were applied were utilized. Using the MPP software, prior to PCA analysis, markers were individually mean-centered and variance-scaled. PCA was performed on the transformed dataset, and the results are presented as a scores plot of the first two principal components (PCs) and a loadings plot to elucidate the contribution of each marker to PC positioning. PCA was not performed on the RG3 taxa due to the limited number of acquired samples.

## Supplementary information

Supplementary information.

## Data Availability

The datasets generated and analyzed during the current study can be reproduced from the raw metabolomic data files that have been deposited to the EMBL-EBI MetaboLights database under the identifier MTBLS1737. The complete dataset can be accessed at https://www.ebi.ac.uk/metabolights/MTBLS1737.
