# Supplementary material for: Metabolic Profiling of Volatile Organic Compounds (VOCs) Emitted by the Pathogens Francisella tularensis and Bacillus anthracis in Liquid Culture
Source: Sci Rep. 2020 Jun 9;10:9333. doi: 10.1038/s41598-020-66136-0 (PMC7283342; doi:10.1038/s41598-020-66136-0)
Supplement: Supplementary file 1 — Supplementary information. [file 41598_2020_66136_MOESM1_ESM.docx]

**SUPPLEMENTAL INFORMATION**

**Metabolic Profiling of Volatile Organic Compounds (VOCs) Emitted by the Pathogens *Francisella tularensis* and *Bacillus anthracis* in Liquid Culture**

**Authors and Affiliations:** Reese, Kristen L.^1,2^; Rasley, Amy^1^; Avila, Julie R.^1^; Jones, A. Daniel^2,3^; Frank, Matthias^1^*

(1) Biosciences and Biotechnology Division, Lawrence Livermore National Laboratory, 7000 East Avenue, Livermore, CA 94550;

(2) Department of Chemistry, Michigan State University, 578 S Shaw Lane, East Lansing, MI 48824;

(3) Department of Biochemistry and Molecular Biology, Michigan State University, 603 Wilson Rd, East Lansing, MI 48823

***Corresponding Author**: Matthias Frank, frank1@llnl.gov

**Supplemental Figures**


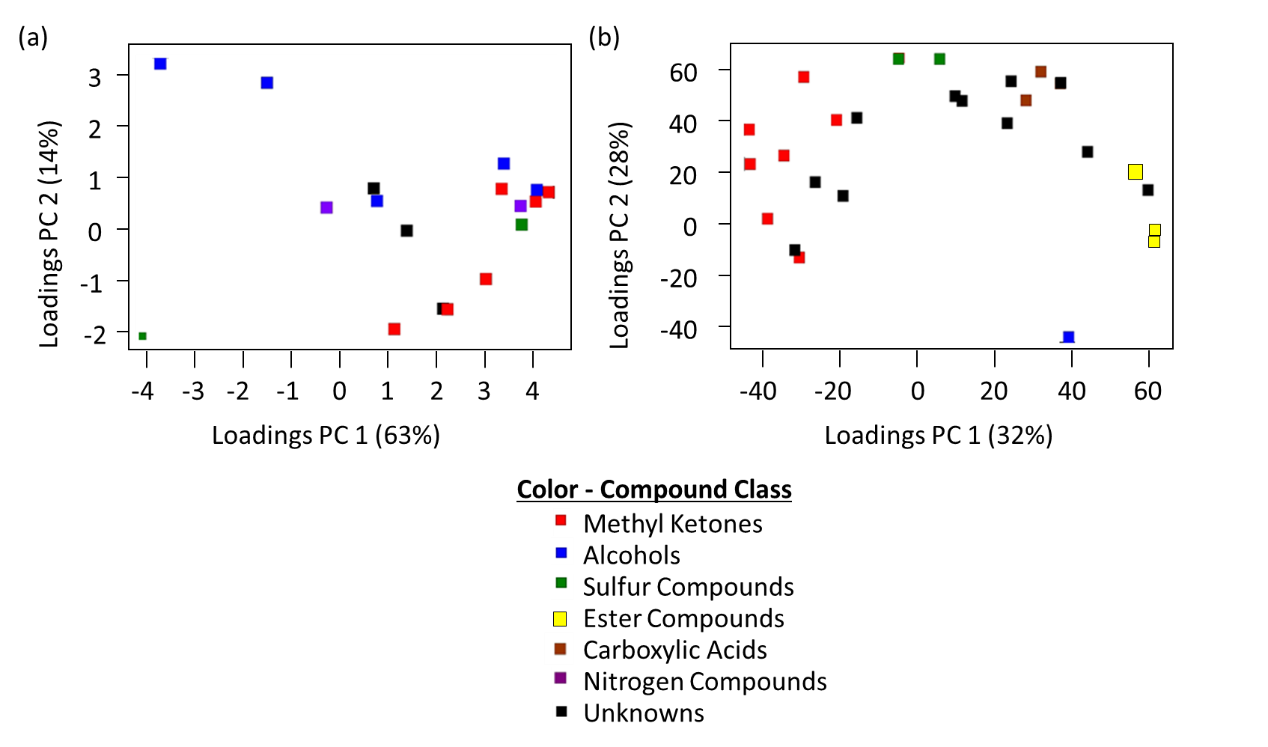


Supplemental Figure 1. PCA loadings plots for VOC profiles of (a) *F. tularensis novicida* and (b) *B. anthracis* Sterne generated using the relative abundances of pathogen VOCs measured using GC-MS across all timepoints (For PCA score plots see Figure 5). Points represent individual VOC markers (colored by compound class) explaining placement of samples on scores plot.

The loadings plots shown in Supplemental Figure 1a and 1b depicts the relative importance of each volatile towards the positioning of individual samples in the scores plots (Figures 5a and 5b, main manuscript). For *F. tularensis* spp. novicida, the first two principal components (PCs) described 63 % and 14 ± % of the variance in data, respectively. Positioning on PC1 is dependent on the abundances of 2,5-dimethylpyrazine and 2-undecanone. Both compounds are present at all timepoints and generally increase with higher timepoints, creating the left-right distribution seen in the scores plot. Positive loading on PC2 indicates a higher percentage of alcohol-containing compounds, indicative of the early log phase and early stationary phase. A progressive shift towards a negative PC2 loading indicates an increasing percentage of methyl ketones, which rise to prominence in the late stationary phase, culminating in the appearance of dimethyltrisulfide. Notably, the volatiles that contributed the most to growth phase separation, those of PC 2, were not the most abundant volatile classes, demonstrating the power of this analysis when characterizing trace compounds in a complex chemical profile. For *B. anthracis* Sterne, the first two PCs described 32 % and 28 % of the variance in data, respectively. Positioning on PC1 in the scores plots exhibits strong dependence on the abundances of 4-heptanol (negative loading) and the sulfur-containing compounds (S-methyl ester butanethioic acid and thiopivalic acid) and carboxylic acids (methylated butanoic acids and 2-ethyl-propanoic acid). A positive positioning on PC2 separates the logarithmic phase, containing a high percentage of esters, from the negatively positioned stationary phase, containing a high percentage of methyl ketones.

**Supplemental Tables**

Supplemental Table 1. Relative abundances of *F. tularensis novicida*-associated VOCs for each replicate at all measured timepoints, separated by growth phase

|  | **Time Post Inoculation (Hours)** | | **0** | **2** | **4** | **8** | **12** | **16** | **20** | **24** | **28** | **32** | **48** | **52** |
| --- | --- | --- | --- | --- | --- | --- | --- | --- | --- | --- | --- | --- | --- | --- |
|  | **Growth Phase** | | **Log Phase** | | | | | | | **Stationary Phase** | | **Decline Phase** | | |
| **Compounds** | **Compound Class** | **Replicate** | **Abundance (Log 10 Values)** | | | |  |  |  |  |  |  |  |  |
| 1-Butanol, 2-methyl- | Alcohols | 1 | 0.00 | 0.00 | 0.00 | 0.00 | 0.00 | 0.00 | 6.72 | 6.65 | 6.82 | 6.83 | 0.00 | 0.00 |
|  |  | 2 | 0.00 | 0.00 | 0.00 | 0.00 | 6.35 | 6.31 | 6.66 | 0.00 | 6.33 | 6.51 | 6.18 | 5.92 |
|  |  | 3 | 0.00 | 0.00 | 0.00 | 0.00 | 0.00 | 0.00 | 6.89 | 6.79 | 6.96 | 6.85 | 0.00 | 0.00 |
| 2-Nonanol | Alcohols | 1 | 0.00 | 0.00 | 0.00 | 0.00 | 6.02 | 6.61 | 6.07 | 5.88 | 0.00 | 0.00 | 0.00 | 0.00 |
|  |  | 2 | 0.00 | 0.00 | 0.00 | 0.00 | 6.06 | 6.60 | 6.10 | 5.87 | 0.00 | 0.00 | 0.00 | 0.00 |
|  |  | 3 | 0.00 | 0.00 | 0.00 | 0.00 | 6.07 | 6.63 | 6.09 | 5.90 | 0.00 | 0.00 | 0.00 | 0.00 |
| Phenylethyl Alcohol | Alcohols | 1 | 0.00 | 0.00 | 5.88 | 6.12 | 6.31 | 6.34 | 6.52 | 6.79 | 6.81 | 6.80 | 6.72 | 6.64 |
|  |  | 2 | 0.00 | 0.00 | 5.79 | 6.03 | 6.27 | 6.35 | 6.47 | 6.70 | 6.68 | 6.83 | 6.66 | 6.72 |
|  |  | 3 | 0.00 | 0.00 | 0.00 | 6.04 | 6.28 | 6.40 | 6.61 | 6.75 | 6.73 | 6.79 | 6.64 | 6.64 |
| 1-Nonanol | Alcohols | 1 | 0.00 | 0.00 | 5.88 | 6.44 | 6.72 | 7.25 | 7.46 | 7.02 | 6.48 | 6.34 | 5.94 | 5.83 |
|  |  | 2 | 0.00 | 0.00 | 5.75 | 6.26 | 6.72 | 7.28 | 7.54 | 7.11 | 6.52 | 6.26 | 5.96 | 5.95 |
|  |  | 3 | 0.00 | 0.00 | 5.76 | 6.33 | 6.67 | 7.24 | 7.35 | 6.69 | 6.26 | 6.09 | 5.76 | 5.76 |
| 2-Undecanol | Alcohols | 1 | 0.00 | 0.00 | 0.00 | 0.00 | 6.13 | 6.34 | 6.26 | 6.16 | 5.97 | 0.00 | 0.00 | 0.00 |
|  |  | 2 | 0.00 | 0.00 | 0.00 | 0.00 | 6.15 | 6.33 | 6.25 | 6.17 | 5.90 | 0.00 | 0.00 | 0.00 |
|  |  | 3 | 0.00 | 0.00 | 0.00 | 0.00 | 6.15 | 6.37 | 6.30 | 6.03 | 6.00 | 0.00 | 0.00 | 0.00 |
| 2-Heptanone | Methyl Ketones | 1 | 0.00 | 0.00 | 0.00 | 6.05 | 0.00 | 6.52 | 6.71 | 6.82 | 6.84 | 6.87 | 6.51 | 6.21 |
|  |  | 2 | 0.00 | 0.00 | 0.00 | 5.74 | 6.18 | 6.46 | 6.73 | 6.82 | 6.68 | 6.88 | 6.46 | 6.38 |
|  |  | 3 | 0.00 | 0.00 | 0.00 | 6.16 | 6.24 | 6.47 | 6.79 | 6.87 | 6.90 | 6.90 | 6.41 | 6.38 |
| 2-Nonanone | Methyl Ketones | 1 | 0.00 | 0.00 | 0.00 | 6.42 | 6.53 | 6.85 | 7.04 | 7.26 | 7.37 | 7.48 | 7.01 | 6.76 |
|  |  | 2 | 0.00 | 0.00 | 0.00 | 6.37 | 6.68 | 6.81 | 7.12 | 7.25 | 7.32 | 7.44 | 6.97 | 6.87 |
|  |  | 3 | 0.00 | 0.00 | 0.00 | 6.52 | 6.58 | 6.85 | 7.17 | 7.41 | 7.52 | 7.51 | 6.89 | 6.91 |
| 2-Undecanone | Methyl Ketones | 1 | 5.17 | 0.00 | 5.90 | 6.15 | 5.96 | 6.04 | 6.64 | 7.09 | 7.20 | 7.26 | 6.28 | 6.06 |
|  |  | 2 | 0.00 | 0.00 | 5.87 | 6.12 | 6.05 | 6.05 | 6.60 | 7.07 | 7.23 | 7.17 | 6.25 | 6.19 |
|  |  | 3 | 0.00 | 5.75 | 5.80 | 6.12 | 5.92 | 5.99 | 6.87 | 7.30 | 7.41 | 7.26 | 6.21 | 6.20 |
| 2-Tridecanone | Methyl Ketones | 1 | 0.00 | 0.00 | 0.00 | 0.00 | 0.00 | 0.00 | 5.96 | 6.82 | 6.94 | 6.97 | 6.09 | 5.92 |
|  |  | 2 | 0.00 | 0.00 | 0.00 | 0.00 | 0.00 | 0.00 | 5.99 | 6.84 | 7.00 | 6.80 | 6.06 | 6.07 |
|  |  | 3 | 0.00 | 0.00 | 0.00 | 0.00 | 0.00 | 0.00 | 6.34 | 7.03 | 7.09 | 6.88 | 6.13 | 6.06 |
| 2-Pentadecanone | Methyl Ketones | 1 | 0.00 | 0.00 | 0.00 | 0.00 | 0.00 | 0.00 | 0.00 | 6.20 | 6.43 | 6.55 | 6.03 | 5.92 |
|  |  | 2 | 0.00 | 0.00 | 0.00 | 0.00 | 0.00 | 0.00 | 0.00 | 6.12 | 6.43 | 6.42 | 5.96 | 6.09 |
|  |  | 3 | 0.00 | 0.00 | 0.00 | 0.00 | 0.00 | 0.00 | 5.43 | 6.34 | 6.62 | 6.55 | 6.10 | 6.08 |
| 2-Heptadecanone | Methyl Ketones | 1 | 0.00 | 0.00 | 0.00 | 0.00 | 0.00 | 0.00 | 0.00 | 5.42 | 5.90 | 5.65 | 5.68 | 5.65 |
|  |  | 2 | 0.00 | 0.00 | 0.00 | 0.00 | 0.00 | 0.00 | 0.00 | 0.00 | 5.69 | 5.53 | 5.57 | 5.78 |
|  |  | 3 | 0.00 | 0.00 | 0.00 | 5.19 | 0.00 | 0.00 | 0.00 | 5.47 | 5.78 | 6.17 | 5.62 | 5.64 |
| Pyrazine, 2,5-dimethyl- | Nitrogen-Containing Compounds | 1 | 0.00 | 6.35 | 6.45 | 6.46 | 6.72 | 7.10 | 7.36 | 7.57 | 7.56 | 7.78 | 7.71 | 7.76 |
|  |  | 2 | 5.72 | 6.39 | 6.36 | 6.30 | 6.86 | 7.24 | 7.44 | 7.71 | 7.44 | 7.66 | 7.82 | 7.78 |
|  |  | 3 | 5.97 | 6.38 | 6.40 | 6.36 | 6.77 | 7.19 | 7.27 | 7.48 | 7.74 | 7.81 | 7.89 | 7.88 |
| 2-Methyl-3-isopropylpyrazine | Nitrogen-Containing Compounds | 1 | 0.00 | 0.00 | 0.00 | 0.00 | 6.11 | 6.21 | 6.45 | 6.74 | 6.90 | 7.04 | 7.08 | 7.00 |
|  |  | 2 | 0.00 | 0.00 | 5.94 | 0.00 | 6.15 | 6.23 | 6.45 | 6.71 | 6.77 | 7.04 | 7.03 | 7.08 |
|  |  | 3 | 0.00 | 0.00 | 0.00 | 0.00 | 6.12 | 6.27 | 6.51 | 6.79 | 6.92 | 7.08 | 7.07 | 7.07 |
| Dimethyl trisulfide | Sulfur-Containing Compounds | 1 | 0.00 | 0.00 | 0.00 | 0.00 | 0.00 | 0.00 | 0.00 | 0.00 | 0.00 | 0.00 | 7.00 | 6.55 |
|  |  | 2 | 0.00 | 0.00 | 0.00 | 0.00 | 0.00 | 0.00 | 0.00 | 0.00 | 0.00 | 0.00 | 6.91 | 6.86 |
|  |  | 3 | 0.00 | 0.00 | 0.00 | 0.00 | 0.00 | 0.00 | 0.00 | 0.00 | 0.00 | 0.00 | 6.74 | 7.01 |
| 1-Propanol, 3-(methylthio)- | Sulfur-Containing Compounds | 1 | 0.00 | 0.00 | 0.00 | 0.00 | 5.87 | 0.00 | 5.88 | 6.04 | 5.93 | 6.11 | 6.09 | 6.05 |
|  |  | 2 | 0.00 | 0.00 | 0.00 | 0.00 | 5.93 | 5.86 | 6.01 | 6.01 | 5.93 | 6.07 | 6.07 | 6.19 |
|  |  | 3 | 0.00 | 0.00 | 0.00 | 0.00 | 0.00 | 6.00 | 6.03 | 6.09 | 6.11 | 6.03 | 6.08 | 6.13 |
| *m/z* 121 _ RI 1002 | Unknown | 1 | 0.00 | 6.19 | 6.19 | 6.29 | 6.07 | 5.98 | 5.99 | 6.15 | 6.23 | 6.30 | 6.22 | 5.99 |
|  |  | 2 | 0.00 | 6.13 | 0.00 | 5.96 | 0.00 | 0.00 | 0.00 | 6.15 | 6.06 | 6.34 | 6.19 | 6.16 |
|  |  | 3 | 0.00 | 6.10 | 0.00 | 5.96 | 6.08 | 5.95 | 6.08 | 6.16 | 6.24 | 6.35 | 6.08 | 6.11 |
| *m/z* 108 _ RI 1049 | Unknown | 1 | 0.00 | 5.91 | 5.67 | 6.39 | 6.53 | 6.48 | 6.54 | 6.67 | 6.62 | 6.70 | 6.62 | 6.55 |
|  |  | 2 | 0.00 | 5.54 | 5.83 | 6.38 | 6.49 | 6.51 | 6.55 | 6.62 | 6.56 | 6.70 | 6.59 | 6.63 |
|  |  | 3 | 0.00 | 5.87 | 5.85 | 6.34 | 6.52 | 6.51 | 6.53 | 6.57 | 6.61 | 6.67 | 6.44 | 6.57 |
| *m/z* 133 _ RI 1110 | Unknown | 1 | 0.00 | 0.00 | 0.00 | 0.00 | 0.00 | 0.00 | 0.00 | 6.22 | 6.09 | 6.03 | 5.92 | 5.83 |
|  |  | 2 | 0.00 | 0.00 | 0.00 | 0.00 | 0.00 | 0.00 | 5.55 | 6.02 | 5.92 | 6.05 | 5.98 | 5.91 |
|  |  | 3 | 0.00 | 0.00 | 0.00 | 0.00 | 0.00 | 0.00 | 0.00 | 5.90 | 6.00 | 6.14 | 5.91 | 5.87 |

Supplemental Table 2. Relative abundances of *B. anthracis Sterne*-associated VOCs for each replicate at all measured timepoints, separated by growth phase

|  | **Time Post Inoculation (Hours)** | | **4** | **8** | **12** | **20** | **24** |
| --- | --- | --- | --- | --- | --- | --- | --- |
|  | **Growth Phase** | | **Log Phase** | | **Stationary Phase** | | |
| **Compound Class** | **Compound** | **Replicate** | **Abundance (Log 10 Values)** | | |  |  |
| Alcohols | 4-Heptanol | 1 | 6.10 | 0.00 | 0.00 | 0.00 | 0.00 |
|  |  | 2 | 6.21 | 0.00 | 0.00 | 0.00 | 0.00 |
|  |  | 3 | 6.12 | 0.00 | 0.00 | 0.00 | 0.00 |
| Carboxylic Acid | Propanoic acid, 2-methyl- | 1 | 0.00 | 5.49 | 0.00 | 0.00 | 0.00 |
|  |  | 2 | 0.00 | 5.62 | 5.93 | 0.00 | 0.00 |
|  |  | 3 | 0.00 | 0.00 | 5.20 | 0.00 | 0.00 |
| Carboxylic Acid | Butanoic acid, 2-methyl- | 1 | 0.00 | 5.82 | 0.00 | 0.00 | 0.00 |
|  |  | 2 | 0.00 | 5.78 | 5.62 | 0.00 | 0.00 |
|  |  | 3 | 0.00 | 6.11 | 5.29 | 0.00 | 0.00 |
| Carboxylic Acid | Butanoic acid, 3-methyl- | 1 | 0.00 | 6.09 | 5.47 | 0.00 | 0.00 |
|  |  | 2 | 0.00 | 6.08 | 5.87 | 0.00 | 0.00 |
|  |  | 3 | 0.00 | 6.27 | 5.58 | 0.00 | 0.00 |
| Ester | Propanoic acid, 2-methyl-, butyl ester | 1 | 6.50 | 6.48 | 0.00 | 0.00 | 0.00 |
|  |  | 2 | 6.53 | 6.59 | 0.00 | 0.00 | 0.00 |
|  |  | 3 | 6.46 | 6.81 | 0.00 | 0.00 | 0.00 |
| Ester | Butanoic acid, butyl ester | 1 | 6.70 | 6.12 | 0.00 | 0.00 | 0.00 |
|  |  | 2 | 6.74 | 6.15 | 0.00 | 0.00 | 0.00 |
|  |  | 3 | 6.66 | 6.35 | 0.00 | 0.00 | 0.00 |
| Ester | Butyl 2-methylbutanoate | 1 | 5.83 | 5.95 | 0.00 | 0.00 | 0.00 |
|  |  | 2 | 5.86 | 6.05 | 0.00 | 0.00 | 0.00 |
|  |  | 3 | 5.75 | 6.26 | 0.00 | 0.00 | 0.00 |
| Ester | Butanoic acid, 3-methyl-, butyl ester | 1 | 5.42 | 6.10 | 4.94 | 0.00 | 0.00 |
|  |  | 2 | 5.48 | 6.17 | 0.00 | 0.00 | 0.00 |
|  |  | 3 | 5.40 | 6.37 | 5.03 | 0.00 | 0.00 |
| Methyl Ketone | Methyl Isobutyl Ketone | 1 | 0.00 | 0.00 | 0.00 | 6.30 | 6.10 |
|  |  | 2 | 0.00 | 0.00 | 5.83 | 6.47 | 6.23 |
|  |  | 3 | 0.00 | 0.00 | 5.93 | 0.00 | 6.07 |
| Methyl Ketone | 2-Hexanone, 5-methyl- | 1 | 0.00 | 0.00 | 6.04 | 6.55 | 6.63 |
|  |  | 2 | 0.00 | 5.40 | 5.90 | 6.63 | 6.96 |
|  |  | 3 | 0.00 | 5.75 | 6.18 | 6.30 | 6.59 |
| Methyl Ketone | 2-Heptanone | 1 | 0.00 | 6.50 | 6.71 | 6.68 | 6.68 |
|  |  | 2 | 5.30 | 6.60 | 6.54 | 6.82 | 7.12 |
|  |  | 3 | 5.30 | 6.64 | 6.68 | 6.52 | 6.72 |
| Methyl Ketone | 2-Heptanone, 6-methyl- | 1 | 0.00 | 0.00 | 6.65 | 6.88 | 6.79 |
|  |  | 2 | 0.00 | 0.00 | 6.56 | 7.03 | 7.28 |
|  |  | 3 | 0.00 | 0.00 | 6.63 | 6.83 | 6.81 |
| Methyl Ketone | 2-Heptanone, 5-methyl- | 1 | 0.00 | 0.00 | 5.66 | 5.99 | 6.04 |
|  |  | 2 | 0.00 | 0.00 | 0.00 | 6.12 | 6.51 |
|  |  | 3 | 0.00 | 0.00 | 5.76 | 5.96 | 5.98 |
| Methyl Ketone | 5-Hepten-2-one, 6-methyl- | 1 | 0.00 | 0.00 | 0.00 | 0.00 | 5.39 |
|  |  | 2 | 0.00 | 0.00 | 0.00 | 0.00 | 5.72 |
|  |  | 3 | 0.00 | 0.00 | 0.00 | 4.81 | 5.21 |
| Methyl Ketone | 2-Heptanone, 4,6-dimethyl- | 1 | 0.00 | 0.00 | 5.27 | 0.00 | 5.46 |
|  |  | 2 | 0.00 | 0.00 | 0.00 | 5.59 | 5.88 |
|  |  | 3 | 0.00 | 0.00 | 0.00 | 5.21 | 5.44 |
| Sulfur containing compound | Butanethioic acid, S-methyl ester | 1 | 0.00 | 0.00 | 5.56 | 5.97 | 0.00 |
|  |  | 2 | 0.00 | 5.48 | 5.38 | 5.88 | 0.00 |
|  |  | 3 | 0.00 | 5.57 | 5.70 | 5.82 | 0.00 |
| Sulfur containing compound | Thiopivalic acid | 1 | 5.08 | 5.41 | 5.49 | 5.99 | 0.00 |
|  |  | 2 | 0.00 | 5.50 | 5.37 | 5.71 | 0.00 |
|  |  | 3 | 0.00 | 5.48 | 5.54 | 5.84 | 4.82 |
| Unknown | m/z 80 _ RI 715 | 1 | 0.00 | 5.82 | 0.00 | 5.76 | 0.00 |
|  |  | 2 | 5.55 | 5.80 | 5.70 | 5.88 | 5.68 |
|  |  | 3 | 0.00 | 5.78 | 5.98 | 5.79 | 0.00 |
| Unknown | m/z 57 _ RI 769 | 1 | 0.00 | 0.00 | 5.02 | 5.05 | 0.00 |
|  |  | 2 | 0.00 | 0.00 | 0.00 | 5.28 | 0.00 |
|  |  | 3 | 0.00 | 0.00 | 0.00 | 5.21 | 0.00 |
| Unknown | m/z 43 _ RI 791 | 1 | 6.95 | 7.17 | 6.89 | 6.29 | 5.92 |
|  |  | 2 | 6.97 | 7.10 | 6.67 | 6.09 | 5.69 |
|  |  | 3 | 6.84 | 7.37 | 6.91 | 6.03 | 0.00 |
| Unknown | m/z 43 _ RI 873 | 1 | 0.00 | 0.00 | 0.00 | 0.00 | 4.92 |
|  |  | 2 | 0.00 | 0.00 | 0.00 | 4.82 | 4.89 |
|  |  | 3 | 0.00 | 0.00 | 0.00 | 0.00 | 4.74 |
| Unknown | m/z 45 _ RI 901 | 1 | 0.00 | 0.00 | 0.00 | 5.41 | 0.00 |
|  |  | 2 | 0.00 | 0.00 | 5.21 | 5.07 | 0.00 |
|  |  | 3 | 0.00 | 0.00 | 5.43 | 5.35 | 0.00 |
| Unknown | m/z 57 _ RI 912 | 1 | 5.63 | 5.67 | 0.00 | 0.00 | 0.00 |
|  |  | 2 | 5.70 | 5.81 | 0.00 | 0.00 | 0.00 |
|  |  | 3 | 5.58 | 5.94 | 5.54 | 0.00 | 0.00 |
| Unknown | m/z 43 _ RI 956 | 1 | 5.11 | 0.00 | 0.00 | 0.00 | 0.00 |
|  |  | 2 | 5.20 | 0.00 | 0.00 | 0.00 | 0.00 |
|  |  | 3 | 5.23 | 0.00 | 0.00 | 0.00 | 0.00 |
| Unknown | m/z 58 _ RI 962 | 1 | 0.00 | 5.82 | 0.00 | 0.00 | 0.00 |
|  |  | 2 | 0.00 | 5.81 | 0.00 | 0.00 | 0.00 |
|  |  | 3 | 0.00 | 5.84 | 0.00 | 0.00 | 0.00 |
| Unknown | m/z 43 _ RI 997 | 1 | 0.00 | 0.00 | 0.00 | 5.18 | 0.00 |
|  |  | 2 | 0.00 | 0.00 | 0.00 | 5.23 | 5.39 |
|  |  | 3 | 0.00 | 5.35 | 0.00 | 0.00 | 5.32 |
| Unknown | m/z 90 _ RI 1005 | 1 | 0.00 | 0.00 | 5.11 | 5.16 | 0.00 |
|  |  | 2 | 0.00 | 0.00 | 5.22 | 0.00 | 0.00 |
|  |  | 3 | 0.00 | 0.00 | 5.31 | 5.18 | 0.00 |
| Unknown | m/z 58 _ RI 1104 | 1 | 5.69 | 5.67 | 5.73 | 0.00 | 5.76 |
|  |  | 2 | 5.65 | 5.87 | 5.60 | 5.61 | 5.94 |
|  |  | 3 | 5.49 | 5.81 | 5.66 | 0.00 | 5.60 |
| Unknown | m/z 83 _ RI 1145 | 1 | 0.00 | 5.05 | 0.00 | 0.00 | 0.00 |
|  |  | 2 | 0.00 | 5.10 | 4.51 | 0.00 | 0.00 |
|  |  | 3 | 0.00 | 5.16 | 4.90 | 0.00 | 0.00 |
| Unknown | m/z 58 _ RI 1554 | 1 | 0.00 | 0.00 | 4.92 | 0.00 | 0.00 |
|  |  | 2 | 0.00 | 5.02 | 5.10 | 0.00 | 0.00 |
|  |  | 3 | 0.00 | 0.00 | 4.97 | 0.00 | 0.00 |

Supplemental Table 3. Bacterial concentrations of *F. tularensis* SCHU S4 in modified Mueller-Hinton media and *B. anthracis* Ames in Brain-Heart Infusion media measured at the VOC sampling time points 6 h and 24 h post-inoculation of cultures. The numbers represent the mean of CFU/mL determined from 2 plate counts for each of the 3 culture replicates (total number of plate counts for each time point = 6), the errors represent standard deviation.

|  | 6 h | 24 h |
| --- | --- | --- |
| *Ft* SCHU S4 | (6.7 ± 1.6 ) * 10^5^ | (6.1 ± 2.9) * 10^5^ |
| *Ba* Ames | (6.7 ± 1.4) * 10^6^ | (2.5 ± 0.4) * 10^7^ |

**Supplemental Protocol**

Solid-Phase Microextraction (SPME) Sampling for Volatile Organic Compounds (VOCs) of Liquid Bacterial Cultures

Lawrence Livermore National Laboratory

Protocol: Rasley-2018-001

Facility/Program: SCA_BSL-2_BSL-3 Global Security

Effective Date: 09/26/2018

Level of Use: Continuous Use

LLNL-MI-791181

This document was prepared as an account of work sponsored by an agency of the United States government. Neither the United States government nor Lawrence Livermore National Security, LLC, nor any of their employees makes any warranty, expressed or implied, or assumes any legal liability or responsibility for the accuracy, completeness, or usefulness of any information, apparatus, product, or process disclosed, or represents that its use would not infringe privately owned rights. Reference herein to any specific commercial product, process, or service by trade name, trademark, manufacturer, or otherwise does not necessarily constitute or imply its endorsement, recommendation, or favoring by the United States government or Lawrence Livermore National Security, LLC. The views and opinions of authors expressed herein do not necessarily state or reflect those of the United States government or Lawrence Livermore National Security, LLC, and shall not be used for advertising or product endorsement purposes.

**Document Revision History:**

| **Revision #** | **Date** | **Author** | **Summary of Changes** |
| --- | --- | --- | --- |
| Initial | 07-03-2018 | Amy Rasley | This SOP was developed to establish the procedure for SPME fiber head-space sampling of liquid bacterial cultures without contamination of the SPME fibers with viable bacteria. |
| Rev 1 | 9/17/2018 | Amy Rasley | Revised to include non-contamination test results when SPME fibers are exposed to fully virulent *Bacillus anthracis*. |
| Rev 2 | 9/26/2018 | Amy Rasley | Revised to include non-contamination test results when SPME fibers are exposed to fully virulent *Francisella tularensis*. |

# 1. Purpose

The purpose of this procedure is to demonstrate that solid-phase microextraction (SPME) fibers are not contaminated with viable agent when exposed to the head space of liquid bacterial cultures during sampling for volatile organic compounds (VOCs) prior to removal of SPME fiber sampling devices to a lower physical containment level [e.g., lower biosafety level (BSL), non-BSL laboratory] for GS/MS analysis.

# 2. Scope

This procedure applies to and has been validated for head space sampling for VOCs using SPME fiber VOC collection devices with the following agents:

- *Bacillus anthracis* Sterne (BSL-2)
- *Francisella tularensis* subsp. *novicida* (BSL-2)
- *Bacillus anthracis* Ames (BSL-3)
- *Francisella tularensis* SCHU S4 (BSL-3)

**Note**: This protocol has been developed and validated using attenuated, select agent-exempt strains in a non-registered BSL-2 laboratory for the purpose of demonstrating that SPME fibers used for head space sampling of VOCs from liquid bacterial cultures are not contaminated with viable agent during the sampling process.

*Prior to removing SPME fibers from any registered BSL-2 or BSL-3 BSAT laboratory, this procedure shall be validated on the fully virulent agents and the SOP updated and approved by the Biosafety Officer (BSO) and Responsible Official (RO).*

As of the current revision, this protocol has been validated for use on the fully virulent *Bacillus anthracis* and *Francisella tularensis*. Validation results indicated that there is no potential for SPME fiber contamination during the sampling process. The current SOP has been updated and approved by the Biosafety Officer (BSO) and Responsible Official (RO).

# 3. Responsibility

***Research Personnel***

Research personnel who will be performing head space sampling using SPME fiber collection devices with any of the above listed agents for which this procedure has been validated must be familiar with and adhere to the exact procedures outlined in this SOP. Any deviation from the written procedure will require re-review and re-validation of the procedure.

All research personnel who will be performing this procedure are required to read, understand and review this procedure with the appropriate PI/RI who developed the procedure and must sign the Training Record in Appendix A prior to performing this procedure.

***Principal Investigator (PI)/Responsible Individual (RI)***

The PI/RI of the protocol is responsible for submitting the written procedure to the RO and BSO for review and approval prior to initiating validation of the procedure. In addition, the PI/RI is responsible for submitting validation data to the RO for review and final approval of the procedure prior to initiating experimental work utilizing this procedure.

The PI/RI is also responsible for training and reviewing this procedure with all personnel who will be performing the validated inactivation procedure.

# 4. Procedure

1. **Preparation of Bacterial Cultures for SPME fiber head-space sampling of VOCs**
   1. Using a sterile, disposable loop, transfer a loopful of frozen glycerol stock of the bacterium onto an appropriate agar medium.
   2. Streak for isolation and incubate (static incubator) at 37°C for 24-28 hours until viable growth of isolated colonies is observed on the plate.
   3. Inoculate 10 ml of broth media with 1-5 colonies of bacteria and incubate with shaking (170 rpm) overnight at 37°C.
   4. Subculture bacteria into 25-50 mL of broth media based on desired optical density (OD) in disposable 250 mL Erlenmeyer flasks with vented caps.
      1. Control Flasks:

- Empty Flask (no media)
- Media Only Flask (no bacteria)
  1. Incubate flasks with shaking (170rpm) at 37°C for various time points (varies by experiment). Maximum bacteria concentrations estimated to not exceed 10^9 colony-forming units (CFUs)/mL.

1. **Head Space Sampling using SPME fibers**
   1. At each specific time point, remove the culture flasks from the incubator and transfer to the biological safety cabinet (BSC).
      1. Allow the culture flasks to sit for a minimum of 30 minutes prior to head space sampling to allow for any potential aerosols generated during the incubation phase to settle.
   2. After 30 minutes, insert the SPME fiber (Supelco Portable Field Sampler, Product #57359-U) through the vent in the cap into the head space above the liquid bacterial culture (see **Protocol Figure 1**). Sampling times vary by experiment. Distance between the fiber tip and liquid culture is approximately 3 inches, minimizing the risk of bacteria transfer.


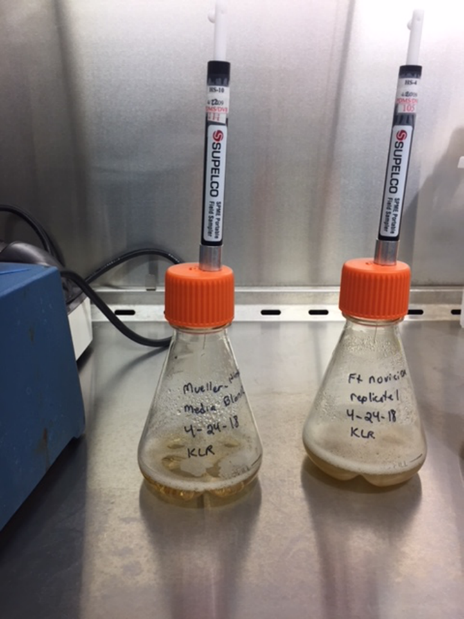
**Protocol Figure 1.** SPME fiber sampling of bacterial culture headspace

- 1. Once head space sampling is complete, retract the SPME fiber back into the sampling device.
     1. Surface decontaminate the exterior of the sampling device prior to removing the device from the BSC.
     2. Place the device in a secondary container for transport out of the laboratory.
        - Surface decontaminate the exterior of the secondary container prior to removal from the laboratory.

**Note:** Refer to **Protocol Appendix B** for procedures and data used to validate that this head space sampling procedure does not result in contamination of the SPME fiber with viable agent.

# 5. Change Control

Revisions to this protocol will be made as necessary. This procedure will be reviewed, and revised as necessary, by the PI/RI, LLNL Responsible Official (RO) and BSO, at least annually or after any change in PI/RI, after any change in the validated procedure and after any failure of the procedure. The PI/RI is responsible for communicating revisions to the appropriate research staff and training the staff on the changes to the procedure.

# 6. Responsible Individual

The PI/RI who authored the procedure is responsible for change control.

# Protocol Appendix A. Training Record

All research personnel who will be performing this procedure are required to read, understand and review this procedure with the appropriate PI/RI who developed the procedure and must sign this Training Record prior to performing this procedure and again after any revision to this procedure.

The PI/RI must submit an electronic copy of the completed training record(s) to the RO who will maintain file copies in the Laboratory Select Agent Program Folder on UCM.

I have read, understood and agree to fully comply with and adhere to the exact procedure, as written in SOP #Rasley-2018-001, Revision 2, *Solid-Phase Microextraction (SPME) Sampling for Volatile Organic Compounds (VOCs) of Liquid Bacterial Cultures* .

| **Name (print)** | **Signature** | **Date** |
| --- | --- | --- |
|  |  |  |
|  |  |  |
|  |  |  |
|  |  |  |

**Protocol Appendix B. Method Validation**

To demonstrate that SPME fibers are not contaminated with viable agent during headspace sampling of liquid bacterial cultures, viability testing was performed on the SPME fibers post-headspace sampling. Viability testing was performed one time using 3 biological replicates for each organism.

1. **Viability Testing Method**
   1. SPME fibers exposed to sampling headspace above liquid bacterial cultures were submerged and agitated in 1 mL of an appropriate growth media for 30 seconds (see **Protocol Figure 2**) and then removed.

- Brain Heart Infusion (BHI) – *B. anthracis* Sterne
- Modified Mueller Hinton – *F. tularensis* subsp. novicida
  1. Media exposed to SPME fibers was incubated (static) for 48 hours at 37°C ±2°C.
  2. Controls:
     1. Broth only (no bacteria) – negative control
     2. 100 µL of inoculum from Replicate #1 in 1 mL media – positive control
  3. After 48 hours, media was observed for growth (turbidity) and results were recorded.
  4. 100% of the media exposed to the SPME fiber was then transferred to the appropriate agar plates (100 µL x 10 plates).
  5. Agar plates were incubated (static) for a minimum of 48 hours at 37°C ±2°C.
  6. After 48 hours, plates were observed for growth.


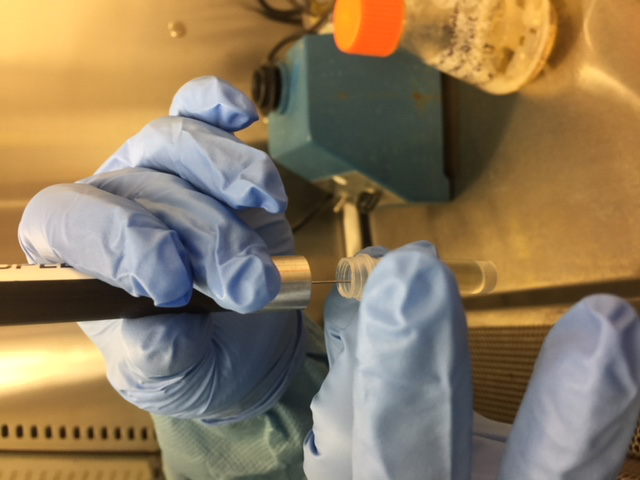
**Protocol Figure 2.** Submerging SPME fiber exposed to headspace above liquid bacterial culture for viability testing.

1. **Results:**

No growth was observed in either the liquid culture exposed to the SPME fibers used to sample the headspace of the tested bacterial cultures for VOCs or when 100% of the exposed media was plated onto agar plates. The negative control (media only, no bacteria) also showed no growth, whereas the positive control (inoculum from replicate #1) showed expected growth indicating that the media uses was capable of supporting the growth of the tested bacteria. These results demonstrate that there is no potential for contamination of the SPME fibers when exposed to the head sampling space of a bacterial liquid culture when following this protocol, as written.
